# Age-dependent changes in phagocytic activity: in vivo response of mouse pulmonary antigen presenting cells to direct lung delivery of charged PEGDA nanoparticles

**DOI:** 10.1186/s12951-024-02743-7

**Published:** 2024-08-12

**Authors:** Emma R. Sudduth, Aida López Ruiz, Michael Trautmann-Rodriguez, Catherine A. Fromen

**Affiliations:** Chemical and Biomolecular Engineering Department, University of Delaware, 150 Academy St, Newark, DE 19716 USA

**Keywords:** Aging, Phagocytosis, Antigen presenting cells, Pulmonary delivery, Pulmonary immunity, Nanoparticles

## Abstract

**Background:**

Current needle-based vaccination for respiratory viruses is ineffective at producing sufficient, long-lasting local immunity in the elderly. Direct pulmonary delivery to the resident local pulmonary immune cells can create long-term mucosal responses. However, criteria for drug vehicle design rules that can overcome age-specific changes in immune cell functions have yet to be established.

**Results:**

Here, in vivo charge-based nanoparticle (NP) uptake was compared in mice of two age groups (2- and 16-months) within the four notable pulmonary antigen presenting cell (APC) populations: alveolar macrophages (AM), interstitial macrophages (IM), CD103^+^ dendritic cells (DCs), and CD11b^+^ DCs. Both macrophage populations exhibited preferential uptake of anionic nanoparticles but showed inverse rates of phagocytosis between the AM and IM populations across age. DC populations demonstrated preferential uptake of cationic nanoparticles, which remarkably did not significantly change in the aged group. Further characterization of cell phenotypes post-NP internalization demonstrated unique surface marker expression and activation levels for each APC population, showcasing heightened DC inflammatory response to NP delivery in the aged group.

**Conclusion:**

The age of mice demonstrated significant preferences in the charge-based NP uptake in APCs that differed greatly between macrophages and DCs. Carefully balance of the targeting and activation of specific types of pulmonary APCs will be critical to produce efficient, age-based vaccines for the growing elderly population.

**Graphical Abstract:**

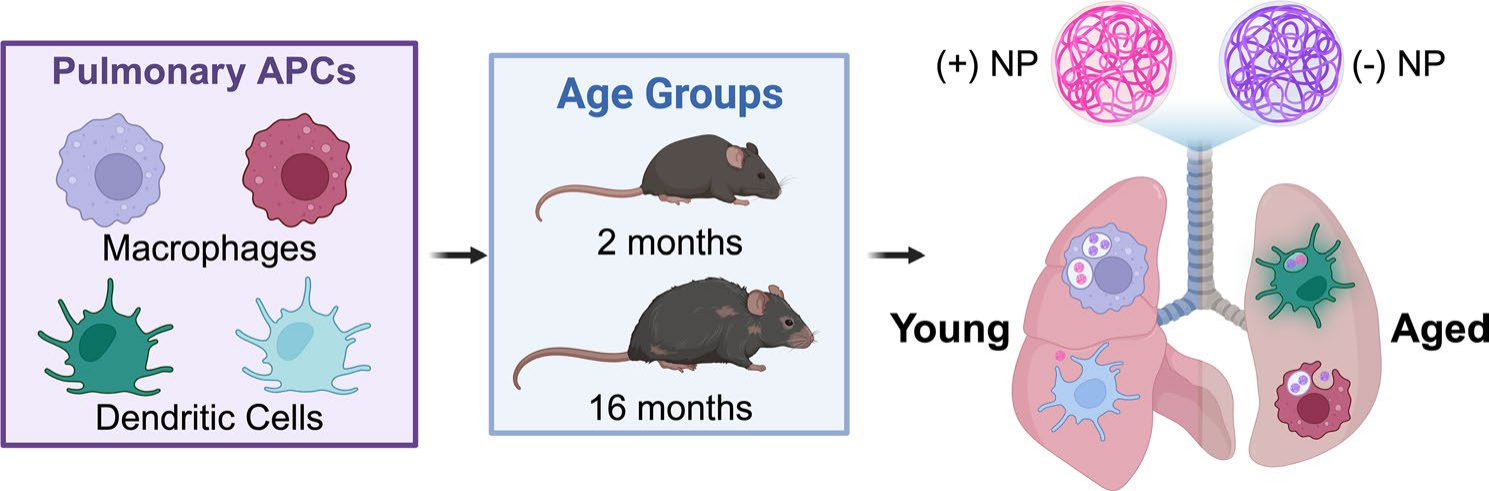

**Supplementary Information:**

The online version contains supplementary material available at 10.1186/s12951-024-02743-7.

## Background

The elderly population is extremely susceptible to severe respiratory infection related to altered immune cell phenotypes in the aged lung [1, 2]. Over time, older individuals demonstrate a reduction in cell-based and other clearance mechanisms in the pulmonary microenvironment, leading to compounding effects influencing immune system regulation [2, 3]. This dysfunction in immune homeostasis leads to a chronic low-grade pro-inflammatory microenvironment in the lung that remains untreated by current therapeutic approaches and unaddressed by existing vaccine platforms [2–4]. It is well established that the altered immune microenvironment in elderly patients leads to reduced immunity and vaccine responses [5–7], as well as decreased therapeutic interactions with immune cells as a result of age [8–10]. Pulmonary delivery may prevail in inducing more effective prophylactics for the elderly, as this route of administration offers a direct approach to target local immune cells in the lung and overcomes challenges of systemic administration that often fails to promote strong cellular or humoral pulmonary responses [8–10]. Indeed, the inherent machinery and function of local pulmonary immune cells to sample inhaled debris make them a ripe target for nano-based designs to activate widespread mucosal immunity [11–14]. However, inhaled delivery has yet to establish age-specific formulations that can address changes in immune function occuring over time [11, 15, 16]. Thus, there is clear motivation to study novel delivery platforms specifically within aged populations to better activate long-term respiratory immunity, which can be accomplished via precise targeting of pulmonary innate immune cells.

Throughout all stages of life, antigen-presenting cells (APCs), a specific class of innate immune cells, act as the first line of active defense against pathogens to clear harmful bodies within the lung [17–19]. These cells are critical for monitoring and maintenance of the lung microenvironment but can additionally activate T and B cell-based immunity, both locally and through drainage into local lymph nodes [19–22]. There exist four primary pulmonary APC types: alveolar macrophages (AMs), interstitial macrophages (IMs), and two types of conventional dendritic cells (cDCs), CD103^+^ and CD11b^+^ DCs [17, 23, 24]. AMs represent the most abundant and widely studied APC in the lung that are uniquely located above the endothelial layer at the air-liquid interface and whose main role is to engulf inhaled debris [9, 19, 20]. IMs are a relatively newly characterized cell type that are less abundant than AMs and located within the interstitium of the lung [20, 23, 25]. Their role is less well-understood but are believed to act as a secondary clearing cell type after AMs and as potential stimulators of local pulmonary T cells [20, 23]. The cDCs are similarly diverse in their roles; CD103^+^ DCs function as the main migratory APC, whereas CD11b^+^ DCs are critical for regulation of local T cell phenotypical expression in the lung [24, 26]. Hence, all four of these cell types are critical players in the immune defense system against major pathogenic infection in the lung, granting them massive unrealized potential as vaccine and drug targets [11, 27, 28]. 

Growing appreciation for the effect of age on the immune system has begun to elucidate the impaired functions adopted by these cell types with increasing age [3, 8, 9]. In the case of lung APCs, there have been a handful of studies that have characterized changes to relative population abundance and an overall decreased uptake of foreign entities in the lung in aged subjects, largely focusing on changes with AMs [3, 29]. Previous work has established that the late stages of aged lungs (murine as well as human) exhibit signs of “inflammaging” (chronic low-grade inflammation associated with age) and “immunosenescence” (altered, less efficient immune response) that contributes to a reduction in APCs’ ability to appropriately target and respond to inhaled pathogenic material [2, 3]. Furthermore, traditional cellular senescence, a state in which cells no longer proliferate and demonstrate increased endosomal activity, can occur in all cell types, further reducing clearance capacity in the lung [30, 31]. Aging is correlated to increasing rates of improper phagocytosis and subsequent immune activation by these cell types over time [2–4, 32]. It is believed that the accumulation of unengulfed debris and dysfunctional cell types within the lung microenvironment inhibits the ability of these APCs to identify and properly respond to pathogenic viruses [8–10]. Particulate-based vaccines capable of targeting pulmonary APCs throughout varied aged environments could offer significant opportunities to overcome these impairments and restore pulmonary health, building on decades of advancements in nanotechnology that can now enable directed immune responses in APCs for prophylactic and therapeutic applications [33–35].

Already, there have been a variety of studies of particulate carriers for pulmonary immune studies, which have been recently reviewed [11]. Importantly, relative size and surface charge of particulates are the major factors that influence their ability to deposit in various lung regions, cross mucosal barriers, and interact with immune cells [11, 36]. However, nanoscale interactions between particles and pulmonary immune cells are still poorly understood and are often times conflicting. For example, inhaled NPs greater than 200 nm are preferentially internalized by AMs and demonstrate reduced mucus penetration capabilities, and yet 200 nm NPs conjugated to antigens have demonstrated greater antibody generation than smaller 30 nm NPs [11, 37]. Moreover, anionic NPs demonstrate greater mucus penetration than positive NPs, and yet AMs preferentially uptake these formulations, while preferential uptake of cationic NPs has been documented in cDCs [11, 38]. Additionally, NP shape and moduli also influence cellular interactions and aerodynamic properties; geometries with higher shape factor have decreased aerodynamic diameters which are capable of deep lung penetration [11, 39]. Even further, material choice and surface properties, such as PEGylation, are intricately involved with not only pulmonary mucosal clearance, but also innate immune responses in the lung [11, 40]. Despite the growing body of work evaluating these design parameters in the lung, most studies have been performed in young murine models, and thus, no systematic approach has been applied to understand the specific changes to APC uptake of NPs in the aged microenvironment. Thus, additional studies are needed to better understand the changing APC immune profile within the lung throughout the aging process to formulate age-specific NP design rules.

To establish NP physiochemical design rules that increase targeting to pulmonary APC subtypes across age, we sought to compare the uptake of poly(ethylene glycol) diacrylate (PEGDA) NPs in varied murine lung APC populations between young and aged mice. We began by characterizing the changes in APC phenotype between young (2-months) and aged (16-months) mice to establish differences in the functionality of these cells and characterize any impairment imparted by age [10, 41]. Then, PEGDA hydrogel NPs of two distinct and opposing charges were synthesized and delivered via orotracheal instillation to both aged groups to quantify aged-based differences in NP phagocytosis as it relates to surface charge. Our results demonstrate that cell counts, NP uptake, and activation profiles of the four APC populations are uniquely affected by age, setting important precedence for the design of future inhalable therapeutics that seek to treat elderly patients.

## Methods

### Materials

For all biological studies, dilutions and formulations were performed with sterile 1X PBS (Phosphate Buffered Saline; Fisher Scientific). All other reagents were used as provided by the manufacturer and stored following manufacturer’s instructions. Reagents were obtained from Fisher Scientific unless otherwise noted within subsections below.

### Nanoparticle synthesis

A pre-polymer solution to form 50 wt% solid PEGDA-based hydrogel NPs was composed of 88.8 wt% PEGDA (Sigma Aldrich), 10 wt% charge-establishing co-monomer, 1 wt% diphenyl(2,4,6-trimethylbenzoyl) phosphine oxide (TPO; Sigma Aldrich), and 0.2 wt% Cy5 Maleimide (Fluoroprobes) in deionized water. 2-aminoethyl methacrylate hydrochloride (AEM, Sigma Aldrich) and 2-carboxyethyl acrylate (CEA, Sigma Aldrich) were used as the cationic ((+)NP) and anionic ((-)NP) functional co-monomers, respectively, to synthesize charged hydrogel NPs. The reverse emulsion photopolymerization technique to synthesize PEGDA hydrogel NPs has been described previously [42–44]. Briefly, polar, pre-polymer solution and non-polar, Silicone Oil AP1000 (Sigma Aldrich) were homogenized at roughly 1 mL total volume using a high-speed, benchtop vortex by placing the tube at an angle and applying pressure continuously along the tube to mix the non-polar and polar solutions. This was followed by tip sonication for 30 s at 30% amplification. Polymerization via UV irradiation was performed using APM LED UV Cube ($\:\lambda\:\:$= 365 nm, distance from light source = ~ 28 cm, intensity = ~ 5–10 mW/cm^2^) for ~ 30 s. Hexanes (Sigma Aldrich) were added to the polymerized mix to wash the silicone oil from the suspension. Particles were then washed twice in ethanol by centrifugation at 13,200 rpm for 10 min and stored in ethanol till use.

### Thermogravimetric analysis (TGA)

Thermogravimetric Analysis (TGA) using TGA 550 (TA instruments) was used to determine bulk stock concentration of NP solutions. A known volume of NP stock solution in ethanol was aliquoted onto a hanging pan and, once loaded into the furnace, the temperature was increased to 90 °C followed by a 10 min isothermal incubation. Final weight measurements from at least two technical replicates were averaged and then divided by the known volume to ascertain the mass concentration.

### Dynamic light scattering (DLS)

Dynamic Light Scattering (DLS) was performed using a Malvern Zetasizer Nano S Instrument (Malvern Instruments). Hydrodynamic diameter (D_h_), polydispersity index (PDI), and zeta potential were measured at room temperature from 0.1 mg/ml solutions of NPs in either water or ethanol. Previously, it was determined that the (+) NPs yielded more disperse NPs suspensions when in ethanol, while (-)NPs were more disperse when in water [45]; DLS measurements were thus obtained in different solvents according to this preference, with sizing confirmed using cryo-SEM. Measurements were averaged from at least two independent replicates.

### Animal studies

All studies involving animals were performed in accordance with National Institutes of Health (NIH) guidelines for the care and use of laboratory animals and approved by the Institutional Animal Care and Use Committee (IACUC) at the University of Delaware. Female C57BL/6 (Jackson Laboratories) of two age groups (2-months and 16-months) were housed in a pathogen-free facility at the University of Delaware and given unrestricted access to chow and water. Mice were dosed via orotracheal instillation following a standard procedure by suspension via their incisors and gently grabbing and moving the tongue aside to block the esophagus, allowing direct delivery to the trachea and minimal dosage to the gastrointestinal tract (Supplemental Figure S1A) [38, 46]. Dosages of 100 µg of NPs were administered in sterile 50 µL 1X PBS under anesthesia with isoflurane. All studies including APC uptake, histology, and inflammatory surface marker analysis had an end point of 24 or 72 h. Terminal bronchoalveolar lavage fluid (BALF) was obtained via cannulation of the trachea restrained by a suture tie (Supplemental Figure S1B). Here, 1 mL of sterile 1X PBS was dispensed into the lungs then was withdrawn and stored for cellular imaging.

### APC phenotype and uptake analysis

After BALF collection, the whole lungs from NP-treated and untreated samples were removed. These were physically minced and digested in 1.5 mL of 5 mg/ml Collagenase IV (Gibco) for up to 2 h with an additional 500 µL of Collagenase IV added and pipette mixed after 30 min until a single cell suspension was achieved. The suspension was then filtered through a 70 μm cell strainer and resuspended in 1 mL RBC lysis buffer (Fisher Scientific). Samples were vigorously pipetted for 30–45 s, centrifuged at 500 rpm for 5 min, and then washed twice in 200 µL 1X PBS. Primary antibody staining was applied to the samples beginning with a Zombie Stain (Biolegend) diluted in 1X PBS for 15 min at room temperature followed by a single wash in 1X PBS and an additional wash in FACS buffer (2% Fetal Bovine Serum in 1X PBS; Fisher Scientific). Prior to further antibody staining, whole lung digests were split in half, one half to assess APC populations and the other to assess APC inflammatory response. Depending on the staining group, a combined antibody cocktail solution (refer to Supplemental Table S1) diluted in FACS buffer was applied for 30 min on ice followed by a single wash in FACS buffer and an additional wash in 1X PBS. Samples were fixed for 15 min in 100 µL of 4% PFA buffer (paraformaldehyde in 1X PBS; Fisher Scientific), washed a single time in 1X PBS followed by an additional wash in FACS. Samples were resuspended in 200 µL of FACS buffer and then run with appropriate fluorophore channels (refer to Supplemental Table S1) continuously on the Novocyte Flow Cytometer (Agilent Technologies). In between stains and washes, samples were centrifuged for 5 min at 500 rpm. Cell vs. debris gate was determined by gating samples with high forward-scatter area (FSC-A) and side-scatter area (SSC-A). Singlets were isolated as having a 1:1 ratio of SSC-A and side-scatter height (SSC-H), as this has previously been used as a rough correlation for the spherical, cellular relationship between granularity and diameter [43–45]. CD45^+^ cells were then isolated from a histogram plot. Macrophages were identified as MerTK^+^/ CD64^+^, with further gating identifying AMs as MHC II^−^ / SiglecF^+^ and IMs as MHC II^+^/ SiglecF^−^. DCs were identified from a secondary MerTK^−^/ CD64^−^ gate and selected by high expression of MHC II^+^ and CD11c^+^. The two subtypes were then separated as CD103^+^ DCs or CD11b^+^ DCs. NP uptake was determined as %NP^+^ based on the percentage of events greater in fluorescence intensity than 1% of the untreated population on the NP^+^ channel, as well as through median fluorescence intensity (MFI) of NP^+^ cells on the same channel. An additional inflammatory panel was identified with the same initial steps isolating cells, singlets, and CD45^+^ cells. Then AMs were isolated as CD11c^+^/ SiglecF^+^, while a broad cDC class was identified as CD11c^+^ / SiglecF^−^ / MHCII^+^. These cells were also stained with CD80 and CD86 for surface marker comparison. Cellular counts, MFI on cellular marker channels, and relative gating schematics were used for comparison between groups.

### Histology

Post-euthanasia, representative whole lung samples were filled gravitationally with a mixture of 50:50 PBS: OCT (Optical Cutting Temperature, Fisher Scientific) solution via suspension of the mouse by its incisors and administering solution through a cannula in the trachea. After inflating and securing via a suture tie, the whole chest cavity was carefully removed, placed into a block, and then covered with OCT. The block was placed in an ethanol: ice mixture to rapidly freeze the sample and then was stored at − 80 °C. Tissue sections were achieved at 7 and 10 μm using the Avantik QS12 Cryostat (Avantik) and stored at − 80 °C till staining. Immunohistochemistry was performed using the Sakura Tissue-Tek Genie^®^ Advanced Staining System (Sakura Finetek USA) for CD45 stain. H&E (Hematoxylin & Eosin) staining was performed using Leica Autostainer XL (Leica Biosystems). For immunofluorescence histology, samples were fixed for 10 min in cold acetone followed by two washes in PBS. To prevent nonspecific binding, a FACS buffer was applied for 30 min. Following, primary anti-Hamster CD11c (Invitrogen) was applied to sections at 10 µg/ml and left overnight in a humidified chamber at 4 °C. Afterwards, slides were washed twice in FACS buffer and then stained with Goat anti-Hamster IgG (H + L) FITC (Invitrogen) at 1:100 dilution for 1 h at room temperature. After additional two washes in FACS buffer, DAPI (4′,6-diamidino-2-phenylindole; Fisher Scientific) was applied for 30 min. Following a final wash in FACS buffer, Pro-Long Diamond Antifade (Fisher Scientific) was applied before applying a cover slip. Images were acquired using the Biotek Cytation 5 Cell Imaging Multimode Reader (Agilent Biotek).

### BALF cellular uptake imaging

Following collection, BALF was centrifuged at 500 rpm for 5 min and then supernatant was removed and saved for other analysis. The pellet was resuspended in 500 µL RBC lysis buffer and vigorously pipetted for 30–45 s. After a 1X PBS wash, the pellet was resuspended in 200 µL of FACS buffer and plated onto a glass-bottom 96 well plate. Cells were allowed to settle to the bottom of the plate via incubation for 2 h and then the plate was spun down for 5 min at 500 rpm. The samples were washed once in PBS and then fixed in 200 µL of 4% PFA buffer for 15 min. Following a final wash in PBS, cells were resuspended in 200 µL of FACS buffer and stored at 4 °C until staining. Cells were stained with DAPI for 30 min and Fluorescent Dye 488-I Phalloidin (Fisher Scientific) for 5 min with a wash of 200 µL of 1X PBS in-between and after each stain. Cells were resuspended in 200 µL of FACS buffer and then imaged using Biotek Cytation 5 Cell Imaging Multimode Reader (Agilent Biotek).

### Statistical analysis

GraphPad prism 9 (GraphPad Software Inc.) was used to generate all quantitative figures and perform all statistical analyses. Numerical data is represented as mean ± standard deviation (SD) unless reported otherwise in figure captions. Appropriate *post hoc* statistical tests were reported in figure captions. Results shown other than histology are representative of at least two independent experiments, with particle or biological replicates reported in figure captions.

## Results

### Comparison of immunophenotype between 2- and 16-month murine models

**Fig. 1:**
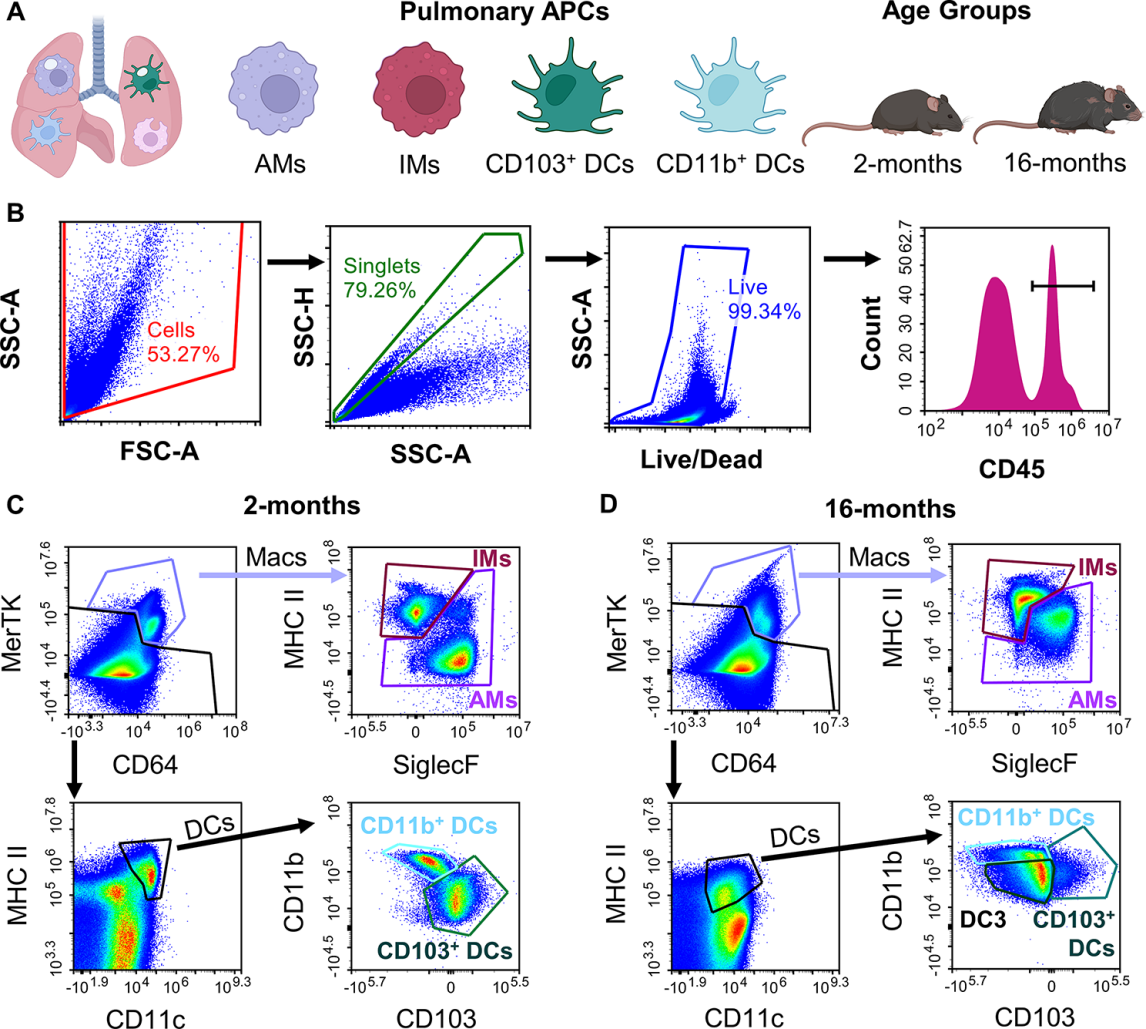
Advanced age demonstrates shift in APC population phenotype. Whole murine lung samples were digested into a single cell suspension for multicolor flow cytometry analysis of four pulmonary antigen presenting cells (APCs) of interest (alveolar macrophages (macs.; AMs), interstitial macrophages (IMs), CD103^+^ dendritic cell (DCs), and CD11b^+^ DCs across two ages (2- and 16-months). (**A**) Study overview schematic including cells and age models of interest. (**B**) Primary gating for cells, singlets, live cells, and CD45^+^ cells. **C**-**D**.) Representative flow gating scheme for 2-months (**C**) and 16-months (**D**)

We first sought to characterize the basal APC phenotype within the 2- and 16-month C57BL/6 mice. We established a flow-cytometry gating scheme of single-cell whole lung digests to identify AMs, IMs, CD103^+^ DCs, and CD11b^+^ DCs in the two age groups (Fig. 1A, staining information Supplemental Table S1) [25]. After gating for cells, followed by singlets, and then the live cell population, the samples were further isolated for myeloid origin through CD45^+^ expression (Fig. 1B). Following this, macrophages could be isolated by high CD64 and MerTK expression, which were then further categorized as AM or IM using SiglecF and CD11b expression. DCs were isolated from their low CD64 and MerTK but high MHCII and CD11c expression. These were then categorized as CD103^+^ or CD11b^+^. However, when applying the same gating scheme to the aged model (16-months), there appeared to be considerable shifts in the markers used for identification of cell types. Macrophages exhibited higher MerTK expression overall and a drastic increase in CD11b expression for the AM population (Fig. 1D). While the DC gate showed similar expression of MHC II and CD11c compared to the young group, the ungated portion of the sample showed an increase in MHC II overall, somewhat hindering the analogous gating scheme for DCs from the young mice (Fig. 1D). Furthermore, the aged model showed a noticeable decrease in CD103^+^ DC events, and the introduction of a new cell type that was neither CD103^+^ or CD11b^+^, labeled as DC3 (Fig. 1D). This DC3 group showed noticeably low expression of CD11b, CD45, and MHC II in comparison to the other DC types (Supplemental Figure S2) but was a unique and separate population from either of the other DC types, as shown through back gating strategies (Supplemental Figure S2). Previous literature has not explored shifts in lung DC populations in aging studies, but as these cell populations in this age group specifically have rarely before been studied, it is not surprising that unique phenotypes may be discovered.

**Fig. 2:**
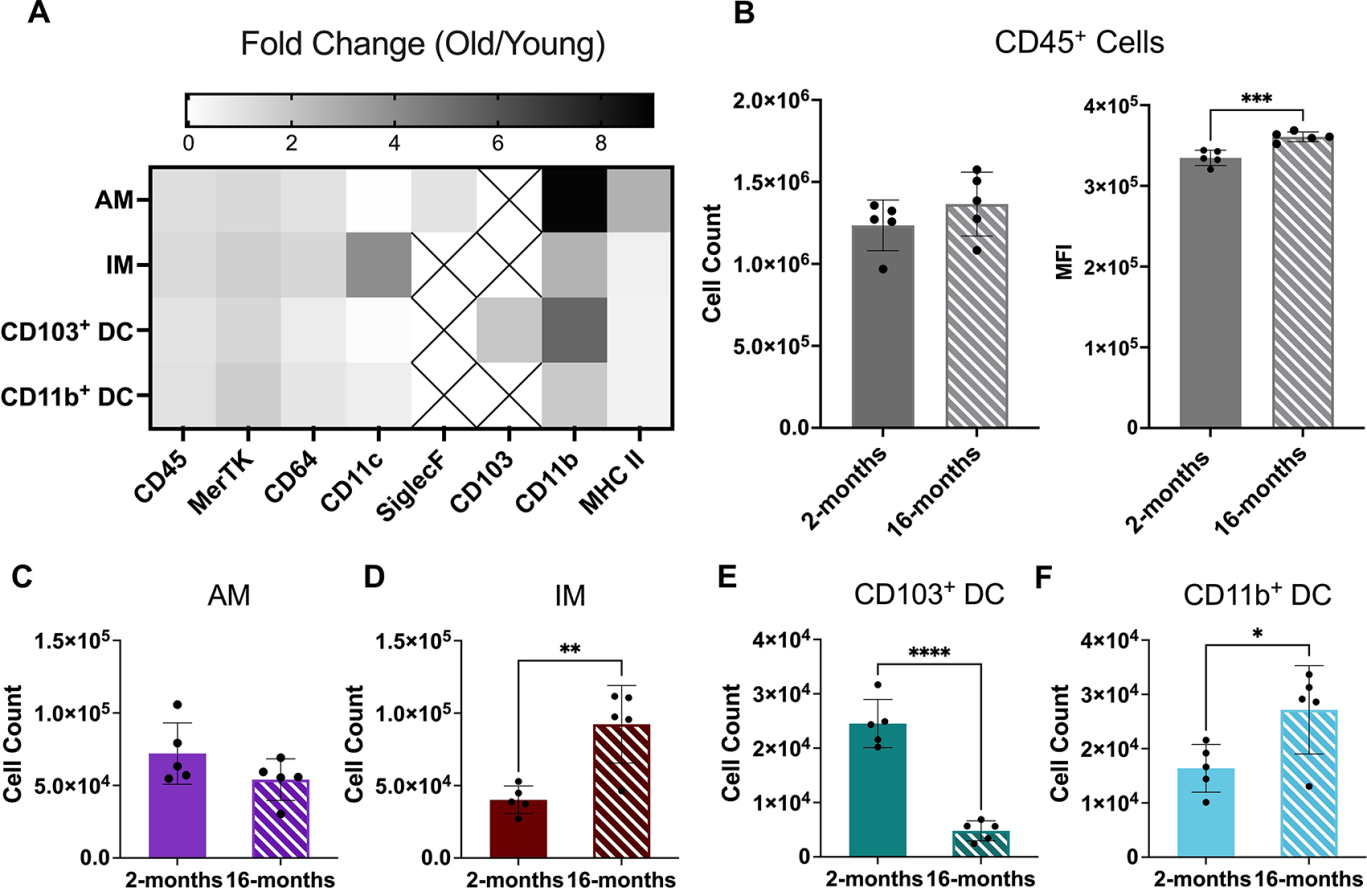
16-month lung presents unique cellular immunophenotype. Cellular comparison was performed of the murine lung for two age groups of mice (2- and 16-months). (**A**) Fold change in surface marker MFI of 16-month isolated cell types compared to average expression of the 2-month group. Boxes with Xs indicate markers that were not expressed in those cell types. (**B**) Multicolor flow cytometry analysis of overall CD45^+^ cell counts and CD45 median fluorescence intensity (MFI). C-F.) Flow cytometry isolated individual cell counts for alveolar macrophages (AM; **C**), interstitial macrophages (IM; **D**), CD103^+^ dendritic cells (DCs; **E**), and CD11b^+^ DCs (**F**) in the two ages. Displayed numerical results represents mean ± SD (*n* = 5) from untreated group results. Indicated significance is calculated via unpaired Student’s t-tests [*p* < 0.05 (*), 0.01 (**), 0.001 (***), < 0.001 (****)]

Population phenotype expression comparison between the two ages showed similar trends in relative expression for the 8 markers selected (CD45, MerTK, CD64, CD11c, SiglecF, CD103, CD11b, and MHC II; refer to Supplemental Figure S3). However, apparent shifts in expression of certain markers were noticeable between the two groups. Thus, relative fold change for each marker of the untreated 16-month group was compared to untreated young mice to characterize changes in phenotype of the four APCs (Fig. 2A). Here, the most significant change observed for all cell types was an increase in CD11b expression, with a nearly 10-fold increase for AMs and 6-fold increase for CD103^+^ DCs (Fig. 2A). AMs also demonstrated a significant increase in MHC II expression compared to other cell types (Fig. 2A). These results for AMs are in line with other previously reported analysis of aged lung models, but as of yet had not been quantified in a pre-senescent, 16-month aged model [47]. Another interesting result was a substantial increase in CD11c expression for the IMs (Fig. 2A). This cell type does not typically present CD11c; however, other studies have found greater presence of CD11c^+^ macrophages in inflamed microenvironments and it is known that this marker is inherently involved in phagocytosis [48–50]. Further quantitative analysis from untreated data showed a slight yet nonsignificant increase in CD45^+^ cell counts but an overall significant increase in CD45 expression in the 16-month age group (Fig. 2B). Representative frozen lung histology further confirmed that CD45 expression increased at the tissue-level using a primary CD45 IHC stain (refer to Supplemental Figure S4 A-D).

Moreover, cell counts for the four APC cells of interest all demonstrated changes with age **(**Fig. 2C-F). Here, the 16-month age demonstrated noticeable yet nonsignificant decrease in AM cell counts accompanied with a complementary significant increase in IM cell counts (Fig. 2C and D). This phenomenon has been observed previously in literature and is believed to be the result of a turnover of guard from AM-dominated clearance in young individuals to a more CD11b^+^ cell type dominated lung population over time in aged lungs [51, 52]. Interestingly, the DC cells also showed a similar trend, in which there was a reduction in CD103^+^ DCs events, previously seen in Fig. 1D, accompanied by an increase in CD11b^+^ DC count, which has yet to be reported in literature (Fig. 2E and F). Additional representative H&E staining of frozen lung tissue demonstrated no significant influx of inflammatory bodies in the 16-month sample compared to the 2-month mice (refer to Supplemental Figures S4E-F).

### Altered in vivo nanoparticle uptake by aged APCs in the lung

**Fig. 3:**
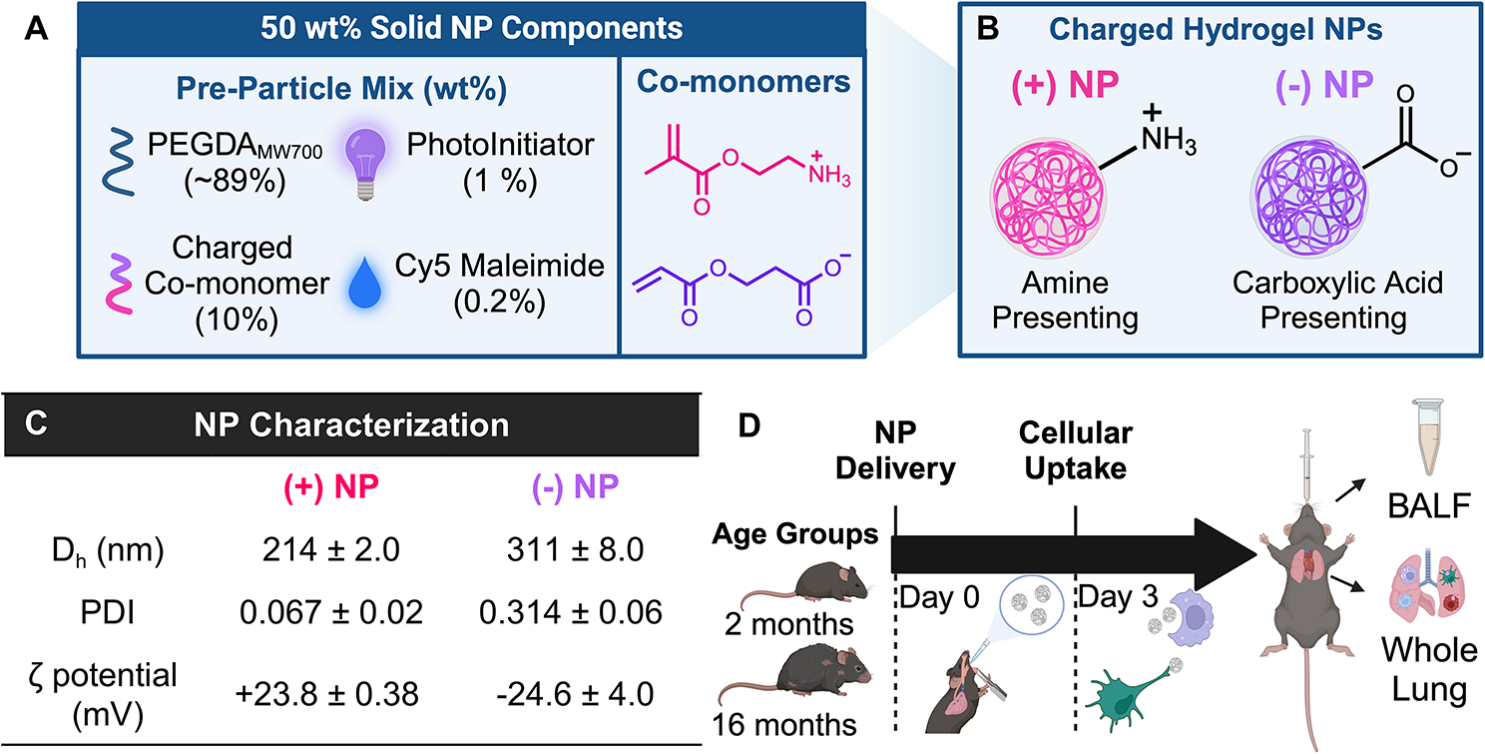
PEGDA Nanoparticle Formulation and Dosing Timeline. Poly(ethylene-glycol) diacrylate (PEGDA) hydrogel nanoparticles (NPs) at 50 wt% solid were synthesized using a reverse emulsion photopolymerization technique for uptake studies. (**A**) Composition of PEGDA pre-particle composition (left) and specific co-monomer chemical structures (right) of PEGDA NPs. (**B**) Surface presentation of synthesized NPs (pink = (+) NP, purple = (-) NP) (**C**) Dynamic light scattering data for NPs. Results represent mean ± SD, *n* = 2 independent batch replicates. D_h_ = hydrodynamic size, PDI = polydispersity index (**D**) Timeline of dosage experiments with NPs leading to collection of bronchoalveolar lavage fluid (BALF), serum, and whole lung tissue for analysis

In order to quantify the change in NP uptake of these unique age groups, an inert PEGDA hydrogel NP was synthesized using a reverse emulsion photopolymerization technique. This particle chemistry has widely been used in literature, as the monomeric components are readily modifiable for tunability of design (Fig. 3A) [38, 42, 44, 45, 53]. Additionally, similar PEGDA NPs synthesized with the same cationic and anionic monomeric components in single-dose, short term studies have shown non-inflammatory responses in the lung, making them ideal vehicles for pulmonary phagocytic studies of APCs without induction of a pro-inflammatory effect [38, 53]. Here, different NPs charge was evaluated to determine preferential uptake between the aforementioned APCs of interest reported in young and old mice. Two formulations of PEGDA NPs were synthesized with opposing surface charges by inclusion of two charged functional monomers (Fig. 3A). The cationic charged functional monomer contained an amine-presenting functional group, which will be referred to as (+)NP and shown in bright pink data figures for the remainder of this article (Fig. 3B). The anionic monomer contained a carboxylic acid-presenting group, referred to as (-)NP and shown as bright purple data figures from hereon (Fig. 3B). The NPs synthesized here displayed high uniformity in hydrodynamic size (D_h_), polydispersity index (PDI), and zeta potential (Fig. 3C). Both formulations achieved NPs around 200–300 nm in D_h_ and an absolute zeta potential around 25 mV (Fig. 3C). Here, it was desired to achieve zeta potentials greater than 20 mV to ensure distinctions in NP charge and sufficient electrostatic interactions between NPs to minimize aggregation over time. While there is roughly 100 nm difference in D_h_ of the two formulations, the (-)NP had a larger PDI pointing to some degree of polydispersity to ensure a reasonable overlap in particle sizes, such that both formulations should be expected to be internalized via phagocytosis. Cryo-scattering electron microscopy images of hydrogel NPs also demonstrated high sphericity of the particles (refer to Supplemental Figure S5). All further studies delivered these PEGDA NPs directly to murine lungs via orotracheal instillation, a pulmonary delivery technique that has shown restricted lung localization [38], and after a specified amount of time, the BALF and whole lung were successfully acquired for analysis (Fig. 3D). We chose to compare NP uptake to an untreated group in both ages rather than a PBS-only, placebo group since previous studies using PBS-only groups have not demonstrated elevated inflammatory signals or soluble factors and thus the untreated group serves as a useful reference for NP distribution [43, 44, 54, 55].

**Fig. 4:**
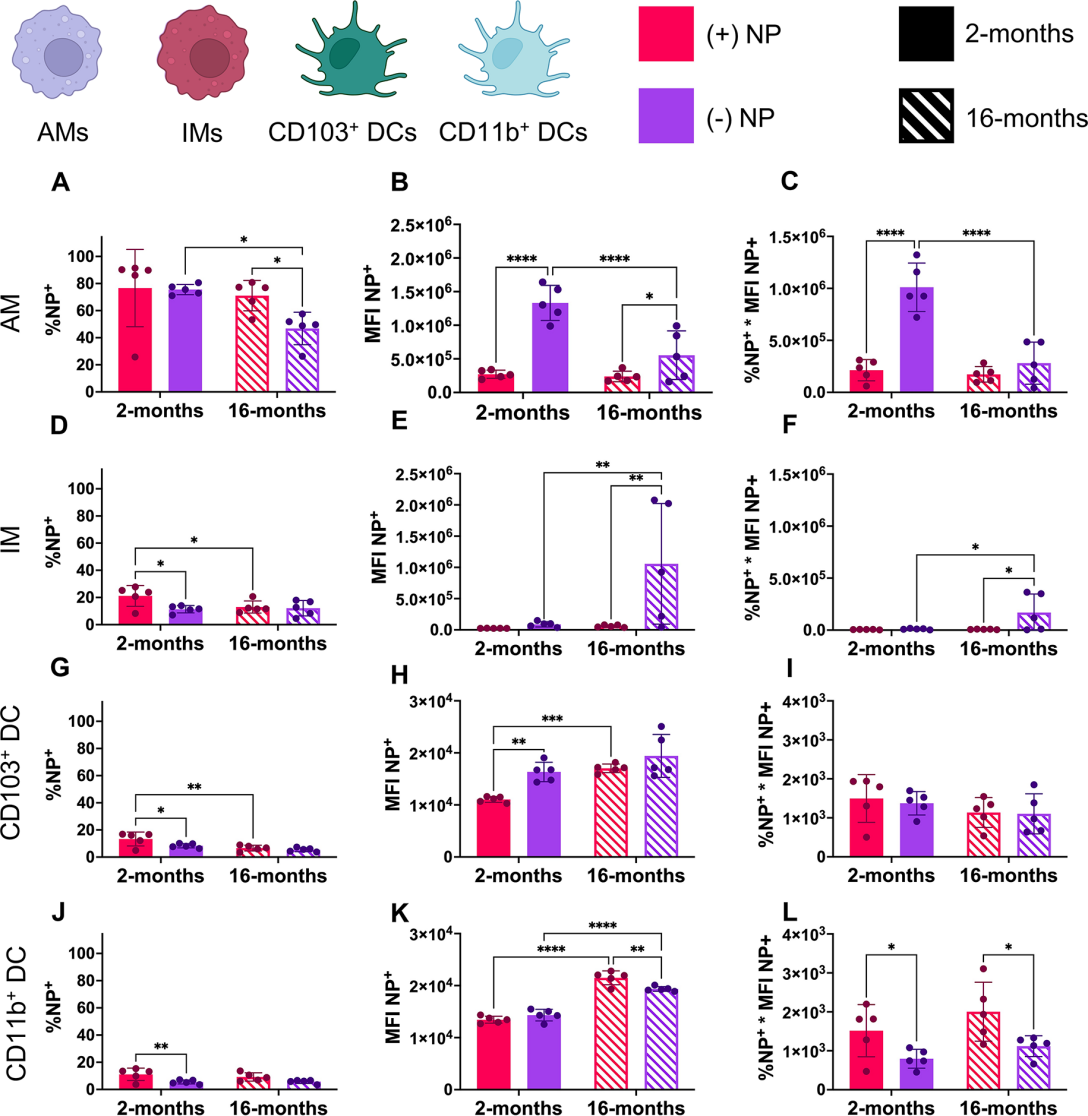
Nanoparticle uptake shifts with charge, age, and type of cell. Two ages of mice (2- and 16- months) were dosed via orotracheal instillation with 100 µg of positive [(+)NP] or negative [(-)NP] PEGDA NPs. After 72 h, flow cytometry determined NP uptake in four APCs in the lung including alveolar macrophages (AMs, **A**-**C**), interstitial macrophages (IMs, **D**-**F**), CD103^+^ dendritic cells (DCs, **G**-**I**), and CD11b^+^ DCs (**J**-**L**). Parameters shown include percent of cell population with NP uptake (%NP; **A**, **D**, **G**, **J**), median fluorescence intensity of the NP channel (MFI; **B**, **E**, **H**, **K**), and the total fluorescence (%NP*MFI; **C**, **F**, **I**, **L**). Data represents mean ± SD (*n* = 5). Indicated significance is calculated via two-way ANOVA [Tukey Test, *p* < 0.05 (*), 0.01 (**), 0.001 (***), < 0.001 (****)]

A preliminary study delivering PEGDA NPs of both charges via orotracheal instillation to 2-month-old murine lungs was used to assess short-term NP uptake after 24 h via %NP^+^(refer to Supplemental Figures S6 and S7). While there was significant uptake of both formulations by the macrophage populations, there was minimal uptake (less than 10%) by DC populations (refer to Supplemental Figure S6). Thus, the remainder of the studies performed used an incubation period of 72 h to allow for appropriate cellular uptake by all populations and to match previously reported literature (Fig. 3C) [38, 54]. From digested lung samples that were treated with either formulation, NP uptake was quantified using %NP^+^(Fig. 4A, D, G and J) as well as median fluorescence intensity (MFI), an average fluorescence value used to roughly determine the quantity of NPs phagocytosed by NP^+^ cells (Fig. 4B, E, H and K). Additionally, a final parameter of total fluorescence that combined these two previously mentioned through multiplication was further used to average NP uptake across these four cells of interest (Fig. 4C, F and I, & 4L).

Of note, AMs showed a significantly higher proportion of NP^+^ cells compared to any other group regardless of age or surface charge (Fig. 4A). This is consistent with known studies of AMs, which have shown that these AMs are the dominating phagocytic cell in the lung [20]. In the 2-month group, the AM population showed nearly 80% uptake after 72 h with no preference to either formulation based on NP^+^ cells (Fig. 4A). However, the aged group showed significant reduction NP uptake, with a reduction between the (-)NP uptake and (+)NP in comparison to their respective young group controls (Fig. 4A). Interestingly, all young groups other than AMs showed a preference for (+)NP uptake, with IMs, CD103^+^ DCs, CD11b^+^ DCs all exhibiting roughly 20% uptake after 72 h of (+)NPs (Fig. 4D, G and J). Other than the CD11b^+^ DC group, the interstitial populations also demonstrated a significant decrease in (+)NP uptake in the aged group but no change in the (-)NP uptake (Fig. 4D, G and J).

While the %NP^+^ uptake exhibited only minor changes across age, more noticeable results emerged for NP^+^ MFI of the four APCs of interest. AMs of both age groups showed high preferential uptake for (-)NPs; however, the aged AM group demonstrated a highly significant (*p* < 0.001) decrease in uptake of (-)NPs **(**Fig. 4B**)**. For the (+)NP, AMs showed no change in uptake across age (Fig. 4B). Additionally, while the IM population showed minimal uptake of NPs in the young population compared to the AMs, there was a dramatic increase (*p* < 0.01) in the quantity of (-)NP uptake in NP^+^ cells in the aged population (Fig. 4E). There was noticeably greater variability within the IM population in terms of (-)NP uptake; this may speak to how undefined and nonlinear the aging process can be between subjects. However, these results are in line with the theory that the IM population may take on a more phagocytic role in the lung with the declining function of the AM population in aged individuals [20, 23]. Furthermore, the combination %NP^+^*MFI for the AM and IM populations supports notion that anionic drug formulations have greater uptake by macrophages in the mucosa (Fig. 4C and F).

In comparison to the dramatic change in uptake of NPs by macrophages, the DC populations overall showed minimal uptake of NPs with overall MFIs roughly 100 times less than that of the AM population (Fig. 4H and K). While both DC types appeared to have a slight trend toward (+)NP preference in %NP^+^, there was significantly higher quantity of (-)NP uptake in the young population of CD103^+^ DCs (Fig. 4H). Interestingly, there appeared to be an increase in NP uptake for all formulations and DC types in the aged group, with CD103^+^ DCs increasing in (+)NP uptake and CD11b^+^ DCs having a drastic increase (*p* < 0.01) in both NP formulations (Fig. 4H and K). Thus, in combination with the %NP^+^ data, both DC populations showed no consistent change in NP uptake with age, while only the CD11b^+^ DC group exhibited preferential (+)NP uptake in both ages (Fig. 4I and L). Additionally, the other DC population isolated, DC3, was analyzed for NP uptake, which showed very similar results to the CD11b^+^ DC population (refer to Supplemental Figure S8). Surprisingly, the DC3 subset showed increased percentage of NP uptake but reduced MFI for NP^+^ cells compared to other DC types (refer to Supplemental Figure S8A-C).

**Fig. 5:**
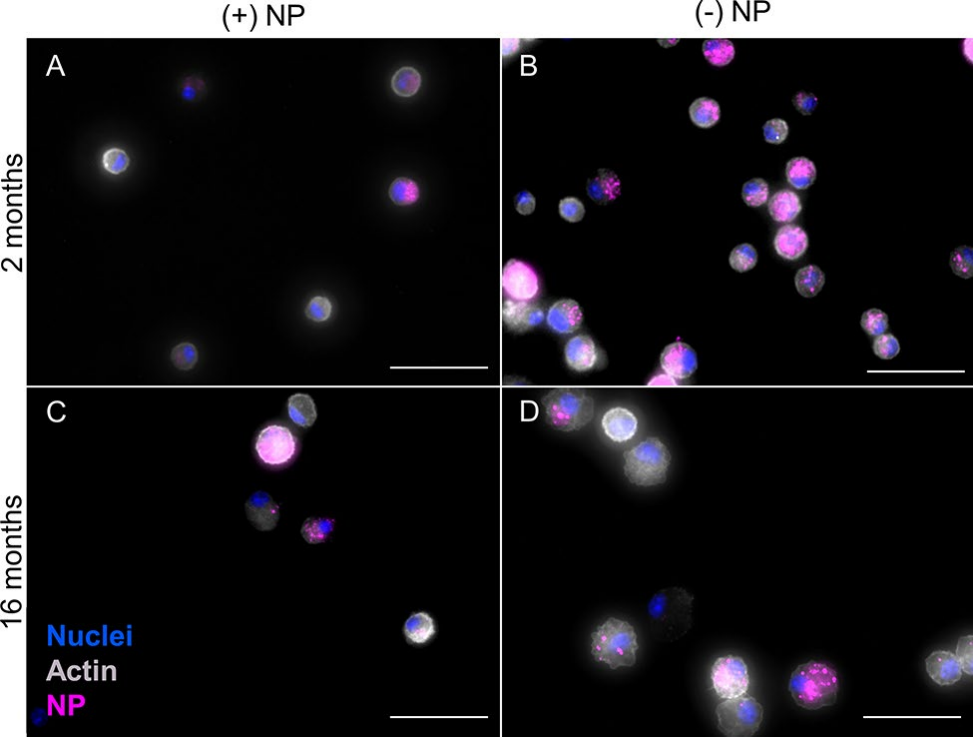
Aged phagocytotic cells in BALF show reduced NP uptake. After 72 h orotracheal instillation of positive (+) or negative (-) PEGDA NPs, cells isolated from BALF from 2- and 16-month mice. Representative stained images to identify nuclei (DAPI; blue), actin (Phalloidin; grey), and NP uptake (pink) were taken for 2-month (+)NP (**A**), 2-month (-)NP (**B**), 16-month (+)NP (**C**), and 16-month (-)NP (**D**). Images were taken using Biotek Cytation 5 Multimode Imager in which exposure was modified to visualize NPs in various samples. 2-month (-) NP samples (**B**) were at least 2x shorter in integration time than all other conditions. Scale bar represents 50 μm

In addition to whole lung flow cytometry results, cells were isolated from bronchoalveolar lavage fluid (BALF) to further visualize changes in NP uptake across age. Thus, cells from BALF were appropriately plated and treated to visualize nuclei (DAPI; blue), actin (Phalloidin; grey), and NPs (pink); Fig. 5). Here, (+)NPs show similar consistent uptake across cells that is similar for both age groups (Fig. 5A and C). In comparison, uptake of (-)NPs in the 2-month group was noticeably higher than the 16-month group (Fig. 5B and D). Additionally, representative images in conjuncture with further supplemental images showed consistent trends within a variety of cells (refer to Supplemental Figure S9). Since BALF has been shown to primarily consist of AMs (> 90%), these results are consistent with flow cytometry results from Fig. 4 [56].

**Fig. 6:**
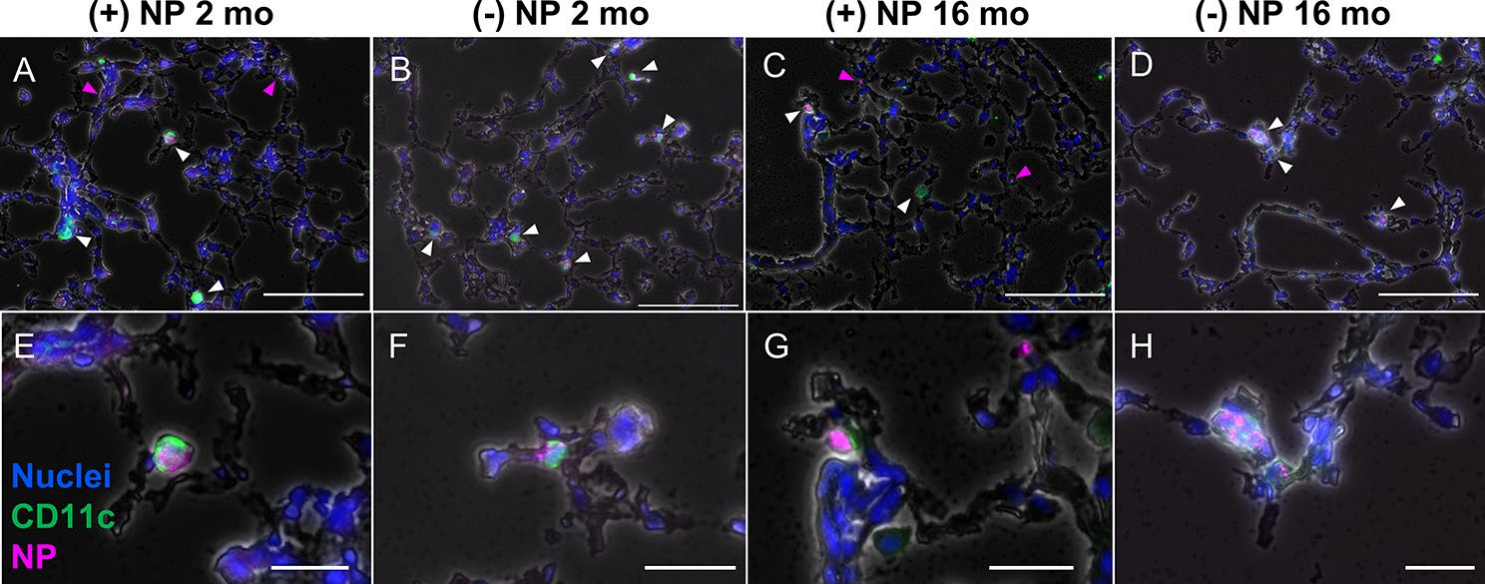
Immunofluorescence histology highlights CD11c^+^cellular uptake of NPs. Frozen whole lung murine tissue was sectioned at 10 μm thickness after 72 h dosage of either positive (+; **A**, **C**,**E**, **G**) or negative (-; **B**, **D**,**F**, **H**) nanoparticles (NPs; pink) via orotracheal instillation. The tissue was stained with primary anti-hamster CD11c, secondary IgG Alexa Fluor 488 (green), and DAPI (nuclei; blue). Representative images (*n* = 1) represent 2-month specimens (A, B, E, F) and 16-month specimens (**C**, **D**, **G**, **H**). White arrows indicate overlap between CD11c^+^, NP^+^ cells, while pink arrows represent non-overlapping “non-localized” NPs. Images acquired using Biotek Cytation 5 Multimode Imager and exposure was modified for viewing of nanoparticles and CD11c stains in each image. Top and bottom row scale bars represent 100 μm and 25 μm, respectively

Further fluorescence histology of frozen lung tissue from representative mice dosed with NPs for 72 h were obtained to ensure trends of cells other than AMs were consistent with flow results. Tissue was treated and imaged to visualize nuclei (DAPI; blue), CD11c (primary anti-hamster CD11c; green), and NPs (pink) Fig. 6). CD11c was used for DC and and to a lesser extent AM isolation. Arrows on individual images point to Cy5-NPs, where white indicates CD11c^+^NP^+^ cells, while pink demonstrates NP^+^ locations without CD11c^+^ cell present. As expected, for both age groups, only the (+)NP formulation showed NPs that were not overlapping with CD11c^+^ cells (Fig. 6A, E, C and G). Since (+)NPs demonstrated much fewer AM-specific uptake and all the interstitial cell types exhibited reduced number of NP^+^ cells in comparison to AMs, this suggests a higher chance of “non-associated” NPs in the tissue, which have not undergone localization specifically with AMs or DCs. In comparison, the (-)NP formulation showed higher consistency in uptake of CD11c^+^ cell across both ages with higher prevalence in the 2-month group (Fig. 6B, F, D and H). Unlike the BALF images however, there was no consistent pattern in the number of NPs that had been phagocytosed between age groups (Fig. 6A and H). This is again consistent with the fact that DCs uptake NPs in low quantities and thus fluorescence imaging may not capture precise changes in this regard. These representative images were consistent with additional supplementary files (refer to Supplemental Figure S10). Thus, these results are also in line with flow cytometry results from Fig. 4.

### Nanoparticle-mediated phenotype of aged APCs in the lung

Once NP uptake was ascertained, the phenotype of the NP^+^ cells was analyzed to determine if the particular advanced age group used here showed signs of increased inflammatory potential. H&E analysis from previous data of 2-month age group dosed with similar PEGDA charged NPs had not shown significant upregulation of inflammatory response [38]. However, it was particularly interesting to ascertain if the contrary would be noted in the 16-month group. Representative H&E images showed no change in inflammatory expression for either formulation for the aged group, signaling that no major event of inflammation occurred due to NP delivery (refer to Supplemental Figure S11). Further analysis of the 8 markers used for identification of the 4 APC cell types showed an increase in CD11b expression for all cells and NP formulations in the aged group after NP uptake, as determined via fold change comparison to MFI of young group for various surface markers (refer to Supplemental Figure S12). Interestingly, AMs from the 16-month group showed only a decrease in CD11c expression after NP uptake while all other cell types showed much greater variation in phenotype following NP uptake (refer to Supplemental Figure S12). Of note, all interstitial cell types showed an increase in MerTK expression and both DC types showed an increase in CD11c following NP uptake compared to the age-matched UT average expression (refer to Supplemental Figure S12).

**Fig. 7:**
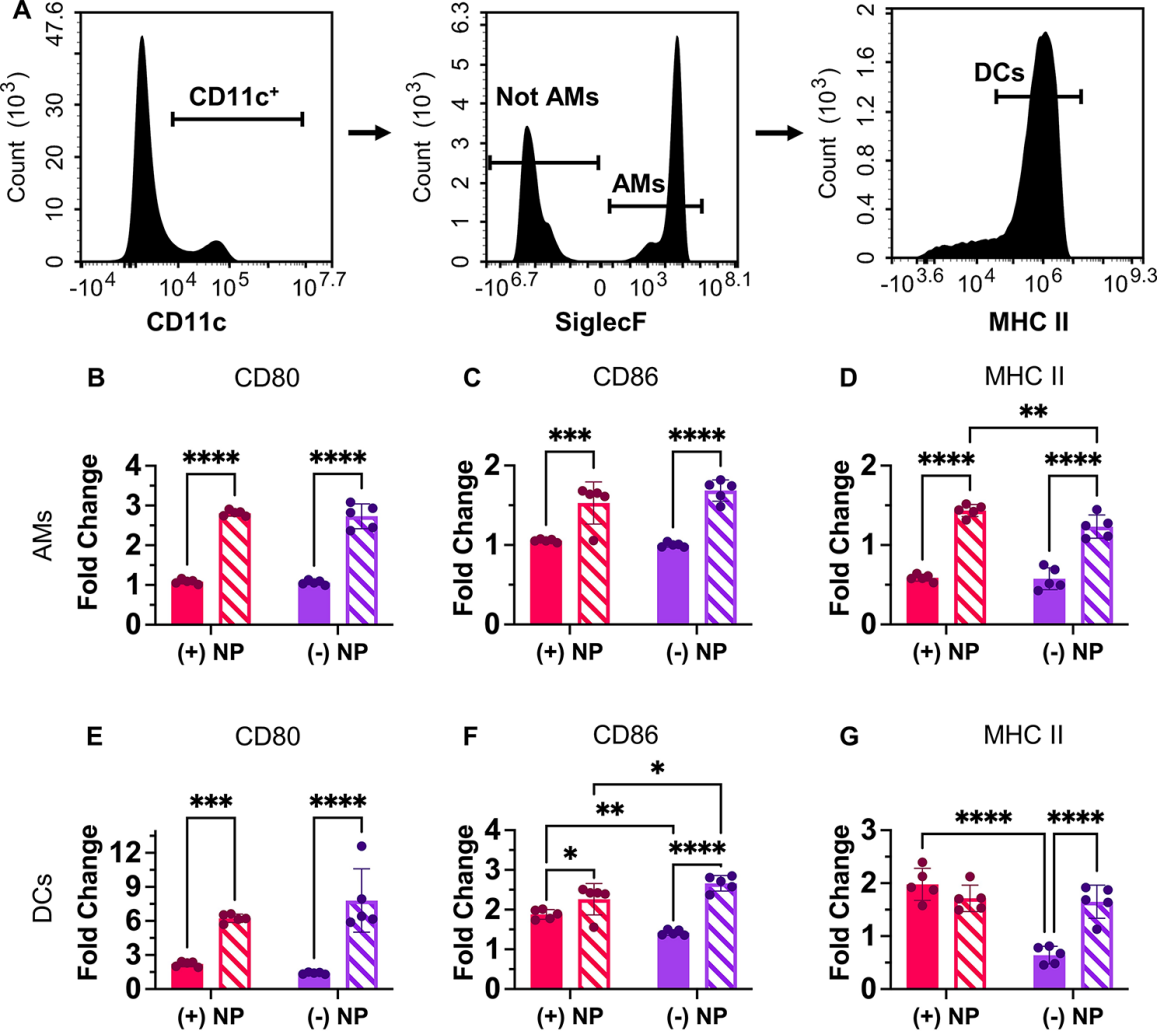
Nanoparticle uptake in aged cells changes activation profile. **A**.) Multicolor flow cytometry results from partial lung tissue digests after 72-hr pulmonary delivery of PEGDA NPs. Alveolar macrophages (AMs; CD45^+^, CD11c^+^, SiglecF^+^) and dendritic cells (DCs; CD45^+^, CD11c^+^, MHCII^high^) were isolated from partial lung digest. AMs (**B**-**D**) and DCs (**E**-**G**) expression of CD80 (**B**, **E**), CD86 (**C**, **F**), and MHCII (**D**, **G**) were compared across NP treatments using fold change from age-appropriate UT. Data represents mean ± SD (*n* = 5). Indicated significance is calculated via two-way ANOVA [Tukey Test, *p* < 0.05 (*), 0.01 (**), 0.001 (***), < 0.001 (****)]

In addition to the full flow cytometry panel used to isolate the 4 APCs of interest in the lung, a simpler panel that used the same initial gating scheme (cells, singlets, live cells, CD45^+^) isolated AMs (CD11c^+^, SiglecF^+^) and broadly DCs (CD11c^+^, SiglecF^−^, MHC^+^) (Fig. 7A). This allowed for additional comparison of pro-inflammatory surface markers CD80 and CD86 as well as MHC II after NP uptake. MHC II, a marker of “self” to immune cells that is critical for antigen presentation to T cells, may become activated after NP uptake and maturation of the cell [21, 43, 54]. The untreated specimens presented different CD80, CD86, and MHC II expression between age groups (refer to Supplemental Figure S13). Results from Fig. 7 represent age-appropriate fold change data from NP^+^ cells of each type allowing for comparison of the increase in inflammatory expression after NP uptake. From this, it was shown that all inflammatory markers for the 16-month AMs showed significantly increased expression compared to the 2-month population for both the cationic and anionic NP formulations (Fig. 7B and D). Additionally, the (+)NP dosed population also exhibited increased MHC II expression in comparison to the (-)NP in the 16-month group AM population (Fig. 7D). Thus, the higher expression of MHC II for (+)NP formulation here is in line with the decreasing rate of (-)NP uptake in the aged AM population.

Similarly, to the AM population, the DCs also showed an increase in CD80 and CD86 expression after NP uptake for each formulation in the aged group (Fig. 7E and F). Of note, in comparison to the AMs, the DCs showed much higher relative fold changes for CD80 and CD86 expression for the age population (Fig. 7E and F). Additionally, both age groups of (+)NP^+^ samples showed a significant increase in CD86 expression in comparison to the (-)NP^+^ population (Fig. 7F). This trend was upheld for the 2-month population for MHCII expression, in which (+)NP formulations were significantly higher than (-)NP expression; however, this trend was not found in the 16-month population (Fig. 7G). However, the increased inflammatory state in the age model may show a trend towards over inflammatory responses by these cell types; thus proper balancing of safe activation of immunity will be necessary for therapeutics designed for the aged.

## Discussion

In this study, we characterized the composition of four pulmonary APC subtypes in mice 2- and 16-months of age and documented changes to their association of NPs of different surface charges, with the goal to develop elderly-specific design rules for pulmonary NP vaccines. We report distinct population changes within the APCs present within the lung in each age group, which have interesting relationships with subsequent NP association that can be extrapolated for better vaccine design. Taken together, these findings illuminate a complex interplay between APC abundance, phagocytic capacity, and NP internalization that is highly dependent on the aged microenvironment and are important to the future of commercial pulmonary vaccine delivery.

Our choice to evaluate 16-month-old mice as our “aged” group roughly corresponds to the age of a ~ 60-year-old human and represents a pre-senescent, yet aged group. This age group in mice has never previously before been characterized for changes in pulmonary APC phenotype; thus, an important goal of our study was to compare the functionality of these cells to those of the young group [10, 41]. Studying pulmonary immune responses to NPs within aged, yet pre-senescent models is critical to learn how clearance mechanisms are altered and thus could be targeted before the onset of significant senescence. In characterizing the APC dynamics, we observe that a 16-month murine model exhibited clear signs of altered APC function in the lung while not demonstrating definitive signs of “inflammaging”. Comparison of phenotypical markers of aging, namely higher expression of CD45, CD11b, and CD11c, agrees with current literature of these APCs in older murine models [2, 3, 47]. Moreover, the enhanced inflammatory reaction toward NP uptake by AMs and DCs as shown through CD80 and CD86 expression highlights the gradual chronic inflammatory environment that is induced in aged individuals, with onsets well before the classical global senescent definition. However, classical inflammation and senescent hallmarks, such as increase in inflammatory bodies, were not observed within the lung tissue [2, 47]. We believe this duality in response can be explained by the fact that, while the mice used here are significantly more aged than the majority of studies on pulmonary immune responses, they are still younger than the senescent benchmark age of 18 months [10, 30]. While senescence certainly alters response and has been linked to the natural degression of aged microenvironment, it is not the first significant change to initiate negative impacts on cellular responses in later stages of life. Thus, a broader, kinetic understanding of natural aging-induced characteristics must be determined to understand the onset of immune impairment and its relationship with vaccine failure in elderly individuals.

In this work, we also elucidated key NP interactions with aged APCs that could be used for elderly-specific pulmonary vaccine designs. Our PEG-based NPs serve as non-inflammatory materials that allow studies of APC tracking within the lung after pulmonary delivery, [53] with important reference data of this chemistry established in the literature for pulmonary APCs in young mice [38, 54]. The NPs of ~ 300 nm enabled rapid phagocytosis by all pulmonary APCs cell types; [33, 34] moreover, the high degree of tunability with this platform makes it a valuable tool to probe age-specific effects [11]. Positive and negative charges on the PEGDA platform were induced via the addition of one of two charge-contributing co-monomers in the pre-polymer monomeric mixture and importantly, neither formulation yielded a global acute inflammatory response in the airway. Notably, variation of the co-monomer led to a nearly 100 nm difference in hydrodynamic diameter between the two groups under the same synthesis conditions. However, both NP types were greater than 200 nm; accordingly, we expect that neither NP formulation would enhance mucus penetration [57], which would have increased their access to the interstitial APCs, and both formulations would be readily internalized by APCs via phagocytosis [58]. However, we do acknowledge that this difference in NP size may have played an additional role in cellular NP uptake or contributed to the variability seen in our results.

Using these NPs, we confirm that AMs demonstrate decreased NP internalization with age, pointing to reduced phagocytic capacity even in this timepoint in the aging process. Interestingly, complimentary increases in the IM population suggest that this cell type can serve as a secondary reservoir of cells capable of phagocytotic function after such reductions in the long-lived AM functionality due to age. Indeed, the aged IMs showed greater phagocytic capacity especially for negatively charged NPs, supporting the role of this cell type, and pointing to a possible alternative APC target for future vaccines for the elderly population. While the mechanism behind this shifted uptake profile remains unknown, changes to the phagocytic capacity of these cells as well as the altered lung microenvironment with age likely contributes. Recent work on the role that (apo)lipoprotein and scavenging receptors have on macrophages responses to PEG NPs and inflammatory diseases may provide necessary answers to the changes observed here [59, 60].

The complimentary shift observed in the DC population numbers also has important ramifications for their use as vaccine targets. Importantly, the increase in CD11b^+^ DC populations could signal an increase in monocyte-derived infiltrates, which occur naturally in aging but are less effective at antigen presentation and migration than CD103^+^ DCs [3, 24]. Additionally, the discovery of a third CD11c^+^/ MHC II^+^ DC type, DC3, was completely unexpected for this study, as no other reports have determined another major class of cDC in the murine lung. However, the majority of current knowledge on aged DCs has been determined using circulating DCs from the blood of aged human subjects and not from lung sources [7, 61, 62]. Moreover, these studies have shown conflicting results on if DC population numbers shift in aged individuals and point to a continued need to advance tissue-specific knowledge of DC behavior and cell populations in aged subjects [7, 61, 62]. The changes we observed in aged DC population dynamics, even in this pre-senescent stage, may explain impairments in antigen presentation that are typically observed in aged subjects and lead to heightened infection and poor response to traditional vaccine treatments.

However, the increased NP^+^ MFI trend by DCs in the aged lung alludes to pulmonary delivery as being a promising alternative route of administration of vaccines for the elderly specifically. Moreover, the knowledge that cationic NPs maintained their preferential uptake in the CD11b^+^ DC type may be helpful in developing vaccines for maturation and even restimulation of local T cells within the lung to fight off reoccurring infections, which are common in the elderly population. The discovery that the DCs, even more so than macrophages, showed a propensity toward over-inflammatory expression after inert NP uptake may serve as a cautionary warning for the use of inflammatory material in elderly-specific vaccines. Hence, while healthy inflammation is necessary to rally other immune cells to the site of infection or disease, there is a delicate balance that must be maintained to stimulate appropriate and transient inflammatory conditions that can boost vaccine-like effects [63–65]. As such, in the chronically inflamed and immunosenescent case of elderly lungs, this balance may be even more difficult, yet critical to ascertain in order to develop prophylactic vaccines that no longer require multiple boosters but rather are specifically designed for expected changes to this microenvironment. Protein- and genetic-level alterations to aged macrophages and DCs in the lung are poorly understood as of now but may explain the differences observed here in APC functionality as it relates to the NP uptake and future vaccine platform designs.

To date, few studies have been performed on aged models due to the high cost of acquiring and maintaining them in healthy condition. Additionally, while chemicals that can induce senescence in certain cell types have been discovered, there is no comprehensive aged in vitro pulmonary model to use for rapid, high throughput testing of immunotherapies for aged macrophages and DCs. Increased access to aged models and development of complex aged in vitro set ups would be extremely useful for studying specific immunoengineering vaccine platform strategies for the elderly population [66]. While insights of age-related decline in adaptive immune function is increasingly understood [2], studies of APC functionality in age are critical in understanding changes to innate immune function in elderly lungs [67, 68]. Our current set of results provides an important outlook to the changes of pulmonary APC phagocytic capacity with advancing age. However, this study is a mere snapshot of an aging, pre-senescent APC response, limited by the selection of a single time point for cargo uptake, a finite number of surface marker utilization, and the comparison of only two distinct age groups in female mice. The work here also does not include any studies of antigen-presentation capacity or migration by these APC types, which is an essential piece of knowledge to understand stimulation of local and distal adaptive immune cells for systemic immunity, especially in response to vaccination. It is pertinent that further studies are performed across a greater variety of age groups, immune cells, and delivery cargos to understand short- and long-term implications of APC immune cell involvement across changes to lifespan that build from this work to fully realize pulmonary vaccines that can better interface with the elderly immune microenvironment of the lung.

## Conclusion

Current vaccine treatment strategies for the elderly have mostly been nonspecific, focusing on high payload delivery and increased frequency to overcome impairments in delivery [7, 69, 70]. Alternatively, potent, targeted inhalable platforms may increase efficacy alongside patient compliance and comfort, as they can be delivered without the use of needles and through handheld devices [11]. Building from the changes observed here in the phagocytic capacity of pulmonary APCs with age and their preference for distinct NP formulation parameters will assist in developing localized vaccines and therapeutics that can promote successful long-term immunity in the aged population. Thus, as the advancing age of our society continuously grows, developing effective vaccines that are tailored to the aged phenotype is critical to ascertain for future generations.

## Electronic supplementary material

Below is the link to the electronic supplementary material.

Supplementary Material 1

## Data Availability

The data sets used and/or analyzed during the current study are available from the corresponding author on reasonable request.

## Abbreviations

APC: Antigen-presenting cell
PEG: Poly(ethylene)-glycol
AM: Alveolar macrophage
DC: Dendritic cell
IM: Interstitial macrophage
IL: Interleukin
TNF: Tumor Necrosis Factor
Mac: Macrophage
NP: Nanoparticle
PBS: Phosphate buffered saline
UV: Ultraviolet
AEM: 2’-aminoethyl methacrylate
CEA: Carboxyethyl acrylate
TGA: Thermogravimetric assay
DLS: Dynamic light scattering
D_h_: Hydrodynamic diameter
PDI: Polydispersity index
NIH: National Institute of Health
IACUC: Institutional Animal Care and Use Committee
BALF: Bronchoalveolar lavage fluid
H: Hours
Min: Minutes
S: Seconds
RBC: Red blood cells
FACs: Flow cytometry buffer
PFA: Paraformaldehyde
MFI: Median fluorescence intensity
OCT: Optical cutting temperature
SD: Standard deviation
H&E: Hematoxylin & eosin
