# Correction: Age-dependent changes in phagocytic activity: in vivo response of mouse pulmonary antigen presenting cells to direct lung delivery of charged PEGDA nanoparticles

**DOI:** 10.1186/s12951-024-03040-z

**Published:** 2024-12-12

**Authors:** Emma R. Sudduth, Aida López Ruiz, Michael Trautmann-Rodriguez, Catherine Fromen

**Affiliations:** Chemical and Biomolecular Engineering Department, University of Delaware, 150 Academy St, Newark, DE 19716 USA

**Correction: Journal of Nanobiotechnology (2024) 22:476**

10.1186/s12951-024-02743-7

Following publication of the original article, an error was identified in the Funding section.

The updated funding is given below and the changes have been highlighted in **bold typeface**.

The authors would like to correct the following two NIH NIGMS awards in the funding section.

(P20GM103446) and (P20GM139760).

The sentence currently reads:

Funding: Research reported in this work was supported by the National Institutes of Health—National Institute of General Medical Sciences under Award Numbers R35GM142866. ES was additionally funded by NIGMS Award Number T32-GM133395. The content is solely the responsibility of the authors and does not necessarily represent the official views of the National Institutes of Health. Microscopy and histology were supported by grants from the NIHNIGMS (P20 GM103446) and (P20 GM139760).

The sentence should read:

Funding: Research reported in this work was supported by the National Institutes of Health—National Institute of General Medical Sciences under Award Numbers R35GM142866. ES was additionally funded by NIGMS Award Number T32-GM133395. The content is solely the responsibility of the authors and does not necessarily represent the official views of the National Institutes of Health. Microscopy and histology were supported by grants from the NIHNIGMS (**P20GM103446**) and (**P20GM139760**).

The original article has been corrected.

