# Supplementary material for: Age-dependent changes in phagocytic activity: in vivo response of mouse pulmonary antigen presenting cells to direct lung delivery of charged PEGDA nanoparticles
Source: J Nanobiotechnology. 2024 Aug 12;22:476. doi: 10.1186/s12951-024-02743-7 (PMC11318229; doi:10.1186/s12951-024-02743-7)
Supplement: Supplementary file 1 — Supplementary Material 1 [file 12951_2024_2743_MOESM1_ESM.pdf]

**Supplemental Information for:**

# Age-Dependent Changes in Phagocytic Activity: in vivo Response of Mouse Pulmonary Antigen Presenting Cells to Direct Lung Delivery of Charged PEGDA Nanoparticles

Emma R. Sudduth, Dr. Aida López Ruiz, Michael Trautmann-Rodríguez, Dr. Catherine A. Fromen*

Chemical and Biomolecular Engineering Department, University of Delaware, 150 Academy St, Newark, DE, 19716, USA

Author ORCIDs:
Sudduth: (0000-0002-8141-6416)
López Ruiz (000-0003-1936-611X)
Trautmann-Rodriquez (0000-0002-2965-3537)
Fromen (0000-0002-7528-0997)

*corresponding author
Catherine A. Fromen
cfromen@udel.edu
150 Academy St.
Newark, DE 19716
(302) 831-3649

| Marker of Interest | Distributor | Fluorophore | Clone | Dilution |
|---|---|---|---|---|
| CD11b | BioLegend | BV711 | M1/70 | 1:100 |
| MHCII (I-A/I-E) | BioLegend | BV785 | M5/114.15.2 | 1:100 |
| CD103 | BioLegend | BV421 | 2E7 | 1:100 |
| Zombie Yellow | BioLegend | Pacific Orange | NA | 1:500 |
| CD45 | BioLegend | FITC | 3—F11 | 1:100 |
| CD64 | BioLegend | PE-Cy7 | X54-5/7.1 | 1:100 |
| MerTK | BioLegend | PE | 2B10C42 | 1:100 |
| CD11c | BioLegend | Alexa Fluor 700 | 3.9 | 1:100 |
| Siglec-F | BD Pharmingen | APC-Cy7 | E50-2440 | 1:100 |
| CD80 | BioLegend | PE/Cy7 | 16-10A1 | 1:100 |
| CD86 | BioLegend | PE | A17199A | 1:100 |
| NP | NA | Cy5 | NA | NA |

**Supplemental Table S1.** Antibody panel for multi-color flow cytometry analysis. Antibodies for staining were purchased through BioLegend or BD Pharmingen with corresponding fluorophore, clone number, and dilution factor listed. Nanoparticles were synthesized with Cy5 marker to capture uptake results, but specific distributor, clone, and dilution factor are not applicable.

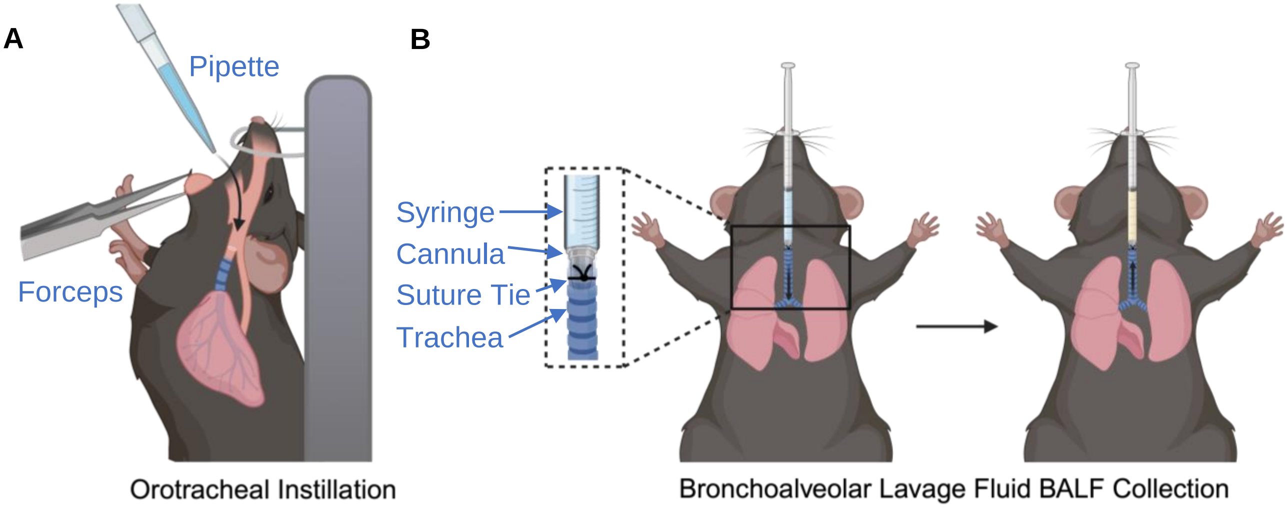


**Supplemental Figure S1: In vivo pulmonary techniques.** Mice were dosed with nanoparticles (NPs) via orotracheal instillation (A), which involves anesthetizing the animal and then hanging by their incisors. Following this, the tongue is gently grasped with forceps and moved to the side of the mouth to restrict swallowing. Then, a pipette is used to deliver 50 µl of desired payload and the nose is covered until audible gasps are heard, indicating successful inhalation of product. After NP delivery, mice are euthanized using $CO_2$ and then they are prepped for bronchoalveolar lavage fluid (BALF) collection (B). This involves pinning the animal on a Styrofoam board on their back to expose their chest and then opening the skin, carefully cutting the diaphragm followed by the ribs to expose the lungs and trachea of the model. Afterwards, a small incision is made in the trachea, above the vocal box, to insert a cannula into the trachea, which is secured with a suture tie around the canula and trachea. Then a syringe filled with PBS can be used to dispense into the lung to wash the cells and lung surface and then retracted to collect the soluble components.

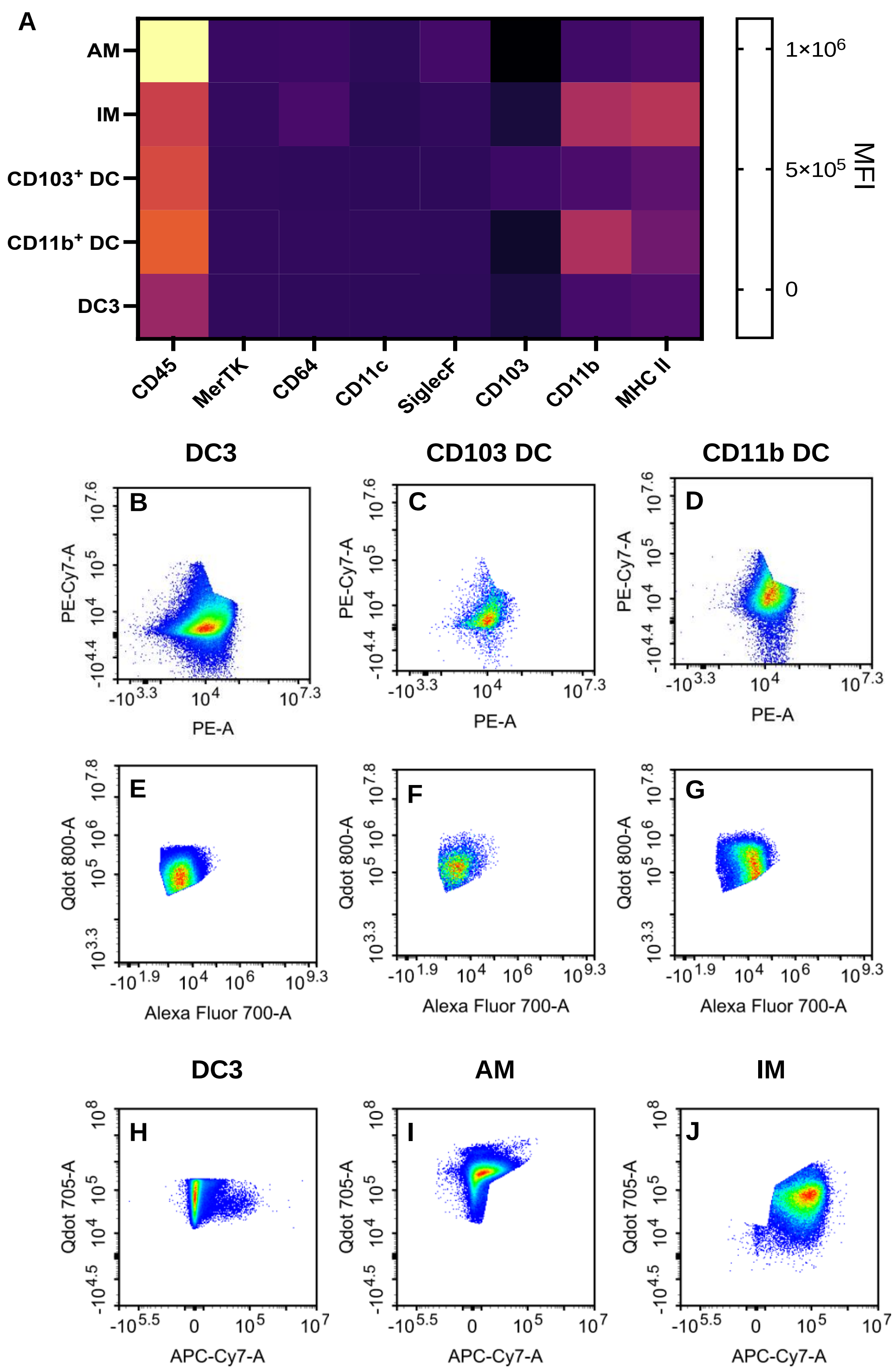


**Supplemental Figure S2: Breakdown of DC3 population phenotype.** A.) Flow cytometry surface marker expression in all cell types for 8 markers used. B-J.) Cell type flow cytometry plots used for isolation of cell types demonstrate specific DC3 phenotype that is unique from other cell types.

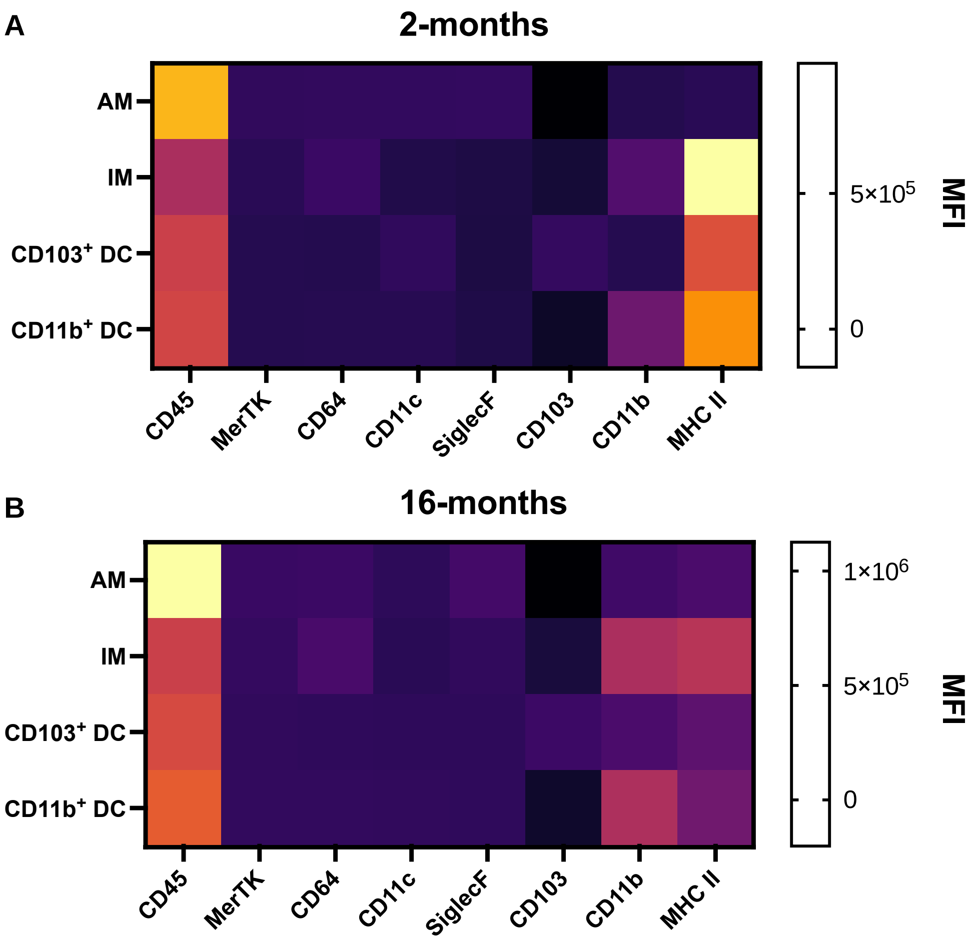


**Supplemental Figure S3. Cell population phenotypes.** Heat map data demonstrates median fluorescence intensity (MFI) for markers used in flow cytometry panel for each of the APCs of interest including alveolar macrophages (AMs), interstitial macrophages (IMs), Cd103+ Dendritic cells (DCs), and CD11b DCs. Data represents untreated specimens of both age groups used in the study (2- and 16-months) with n=5. A.) 2-months. B.) 16-months.

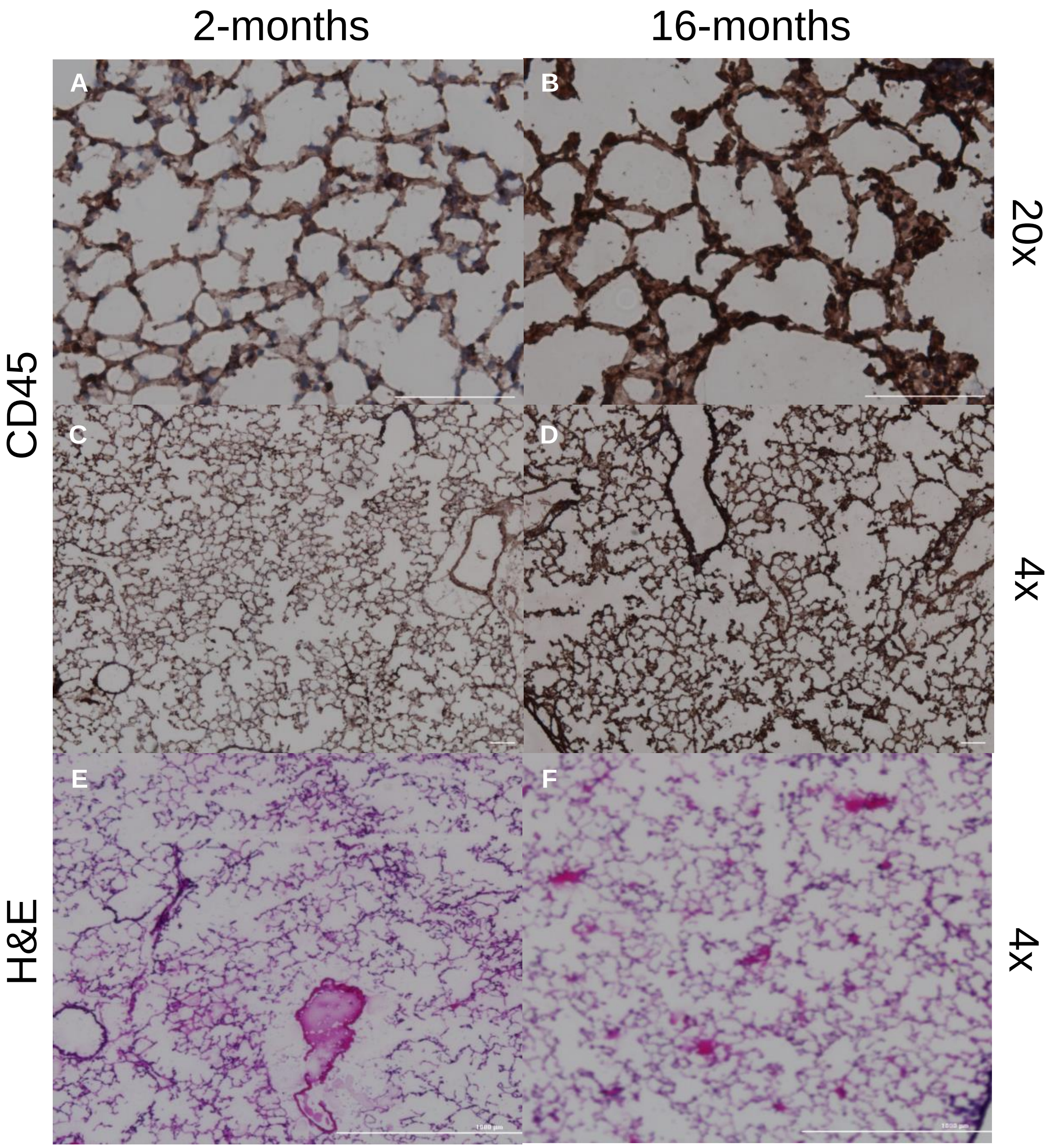


**Supplemental Figure S4: Representative tissue histology for untreated groups.** One representative whole lung sample from each untreated age group (2- and 16-months) was filled with a mixture of OCT and PBS (50:50), removed, rapidly frozen, and then stored at -80 C. Tissue sections at 10 um thickness were acquired and stained with either CD45 (A-D) or H&E (E-F) according to manufacturer’s instructions. A, C, E.) 2-months old. B, D, F.) 16-months old.

(+)NP (-)NP

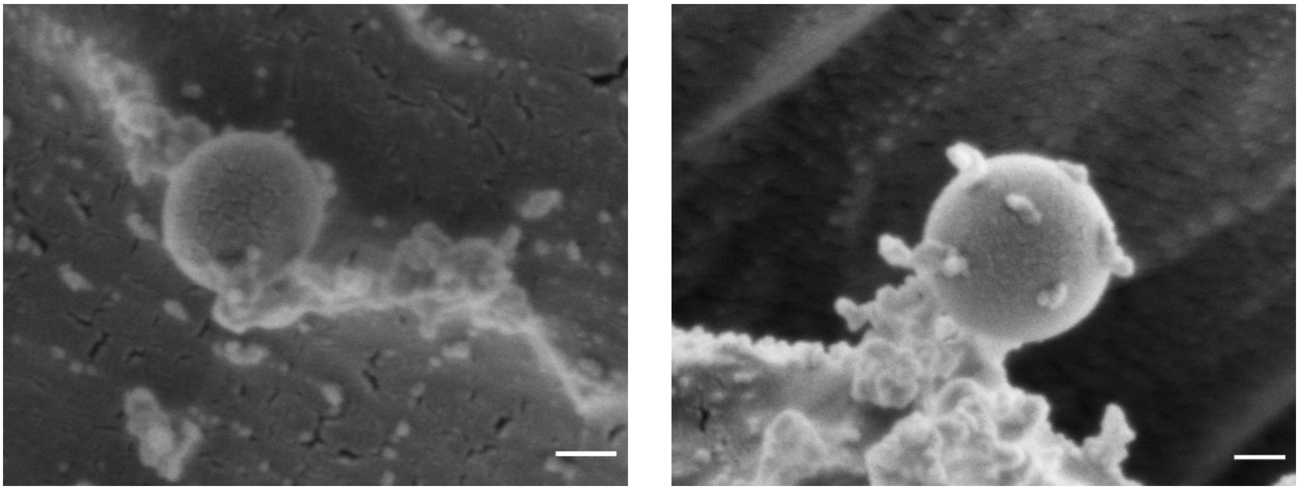

**Supplemental Figure S5. PEGDA Nanoparticle Cryo-SEM.** Poly(ethylene-glycol) diacrylate (PEGDA) hydrogel nanoparticles were characterized previously using cryo-scanning electron microscopy (Cryo-SEM) that demonstrated high sphericity and uniform size. Scale bar represents 100 nm.

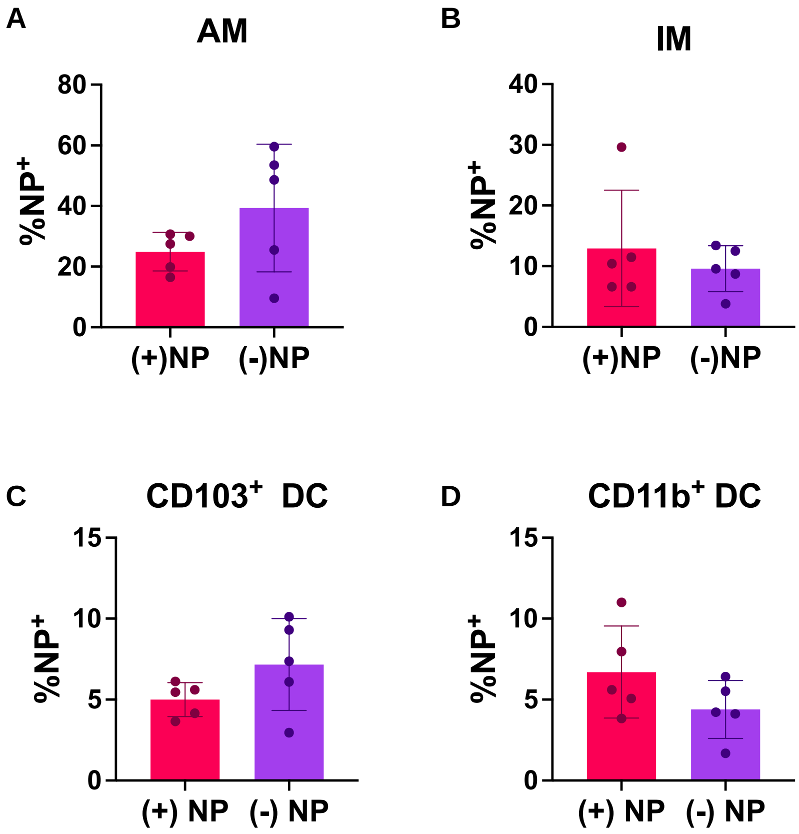


**Supplemental Figure S6. 24 hr timepoint NP uptake in young mice.** 2-month-old mice were dosed orotracheally with PEGDA NPs of either positive [(+)NP] or negative [(-)NP] charge. After 24 hrs, whole lungs were extracted and nanoparticle uptake was determined in four cell types (AMs, IMs, CD103$^{+}$ DCs, CD11b$^{+}$ DCs) per %NP+ gates. Values represent mean ± standard deviation (n=5). T-test analysis determined that all formulations had non–significant changes for each cell type. A.) Alveolar Macrophages (AM), B.) Interstitial Macrophages (IM) C.) CD103$^{+}$ dendritic cells (DC) D.) CD11b$^{+}$ dendritic cells (DC)

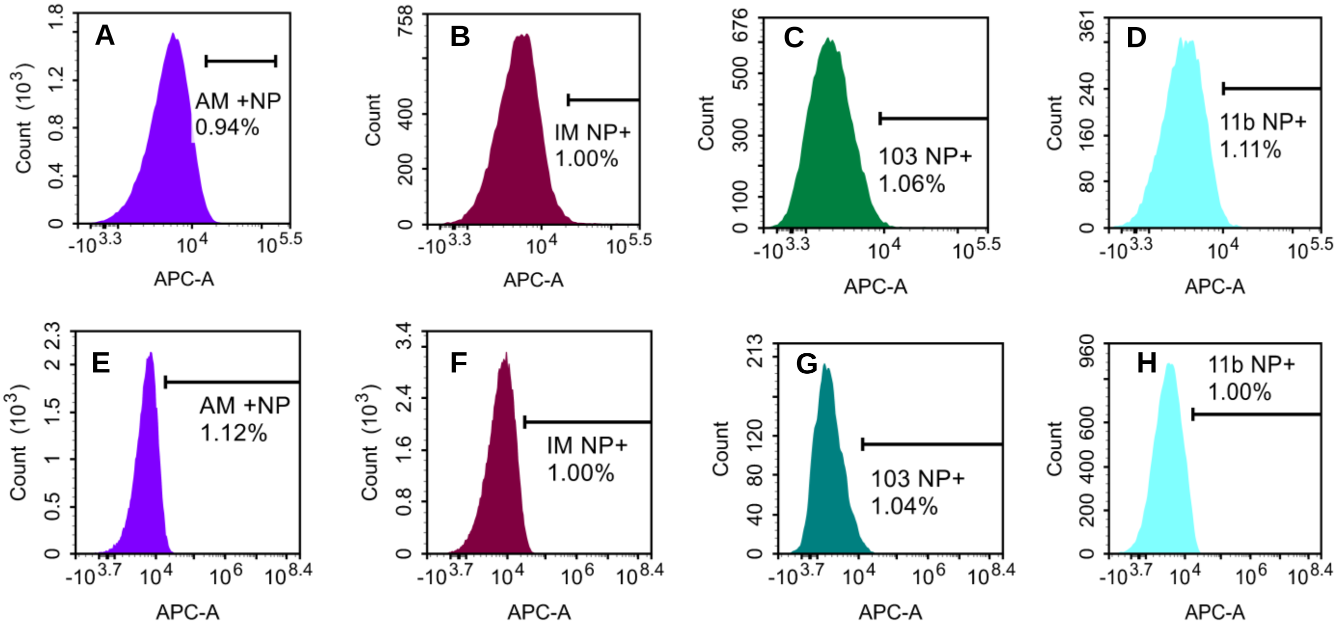


**Supplemental Figure S7. %NP$^+$ gates from untreated mice.** To quantify the percent of the population that had phagocytosed nanoparticles (NPs), %NP$^+$ gates were formed at 1% the untreated population on the channel used to identify nanoparticles (APC). Alveolar macrophages (AMs; A, E) are shown in purple, Interstitial macrophages (IMs; B, F) are shown in dark magenta, CD103$^+$ dendritic cells (DCs; C, G) are shown in green or teal, and CD11b$^+$ DCs (D, H) are shown in light blue. Both age groups (2- and 16-months) used for these studies are presented above. A-D.) 2-months. E-H.) 16-months.

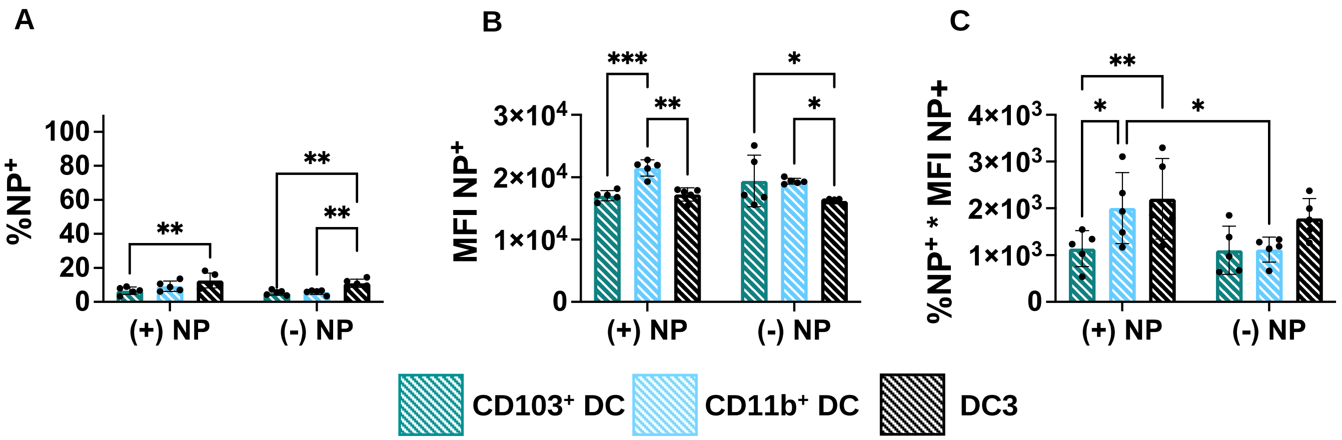


**Supplemental Figure S8: Nanoparticle uptake for additional DC type.** Results are of 72 hr flow cytometry for NP uptake in 16-month DC types. Parameters shown include percent of cell population with NP uptake (%NP; A), median fluorescence intensity of the NP channel (MFI;B), and a multipliable combination of the two parameters (%NP*MFI; C). Data represents mean ± standard deviation (n=5). Indicated significance is calculated via two-way ANOVA [Tukey Test, p < 0.05 (*), 0.01 (**), 0.001 (***), < 0.001 (****)].

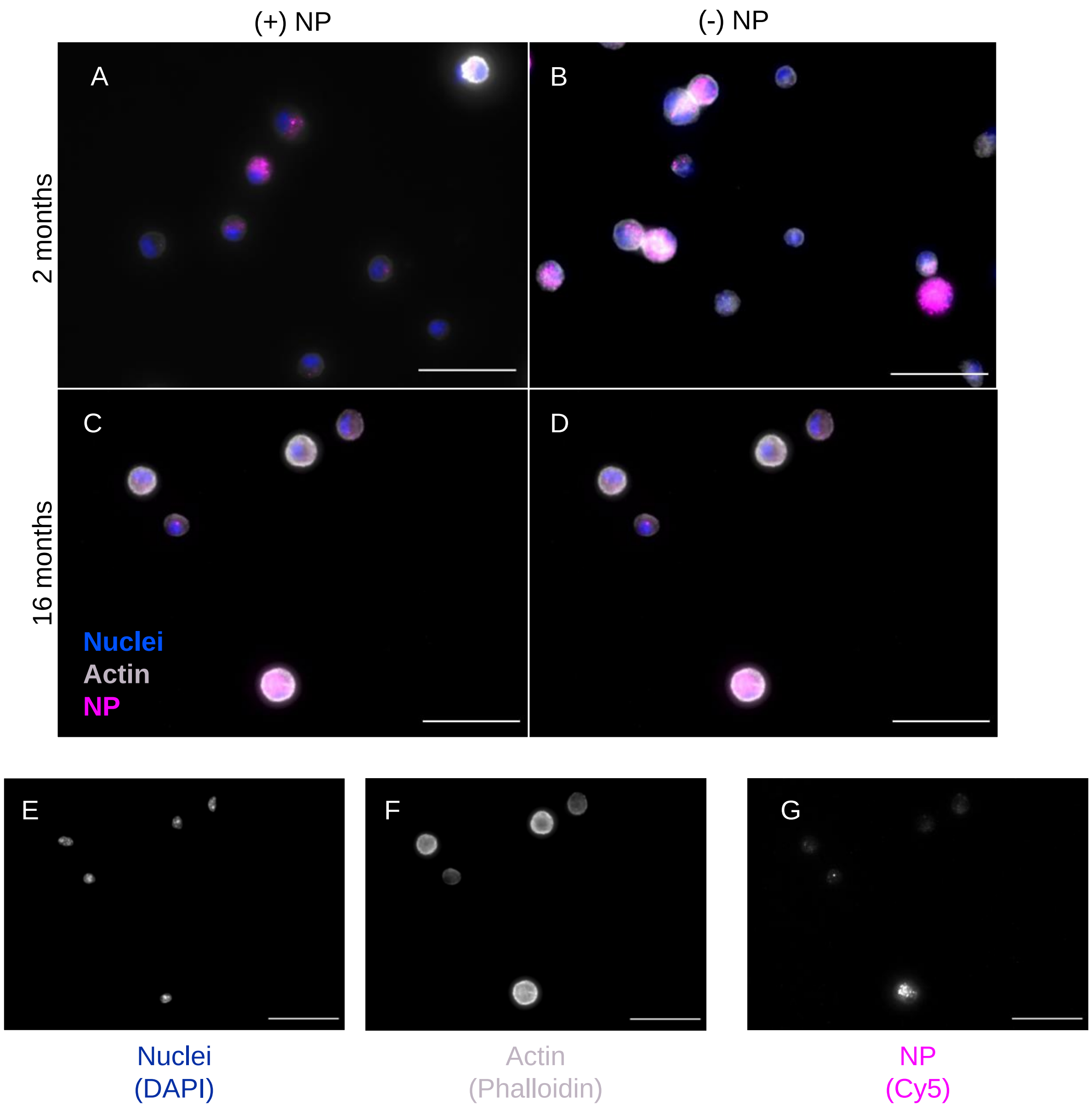


**Supplemental Figure S9: BALF cell replicates and image breakdown.** Nanoparticle-dosed alveolar cells isolated from bronchoalveolar lavage fluid (BALF) from 2- and 16-month mice. Additional representative stained images identify nuclei (DAPI; blue), actin (Phalloidin; grey), and NP uptake (pink). A.) 2-months (+)NP; B.) 2-months (-)NP; C.) 16-months (+)NP; d.) 16-months (-)NP. E-G.) Representative breakdown of composite image including Nuclei (E), Actin (F), and NPs (G). Images were taken using Biotek Cytation 5 Multimode Imager and individually manipulated for viewing of nanoparticles. Scale bar represents 50 µm.

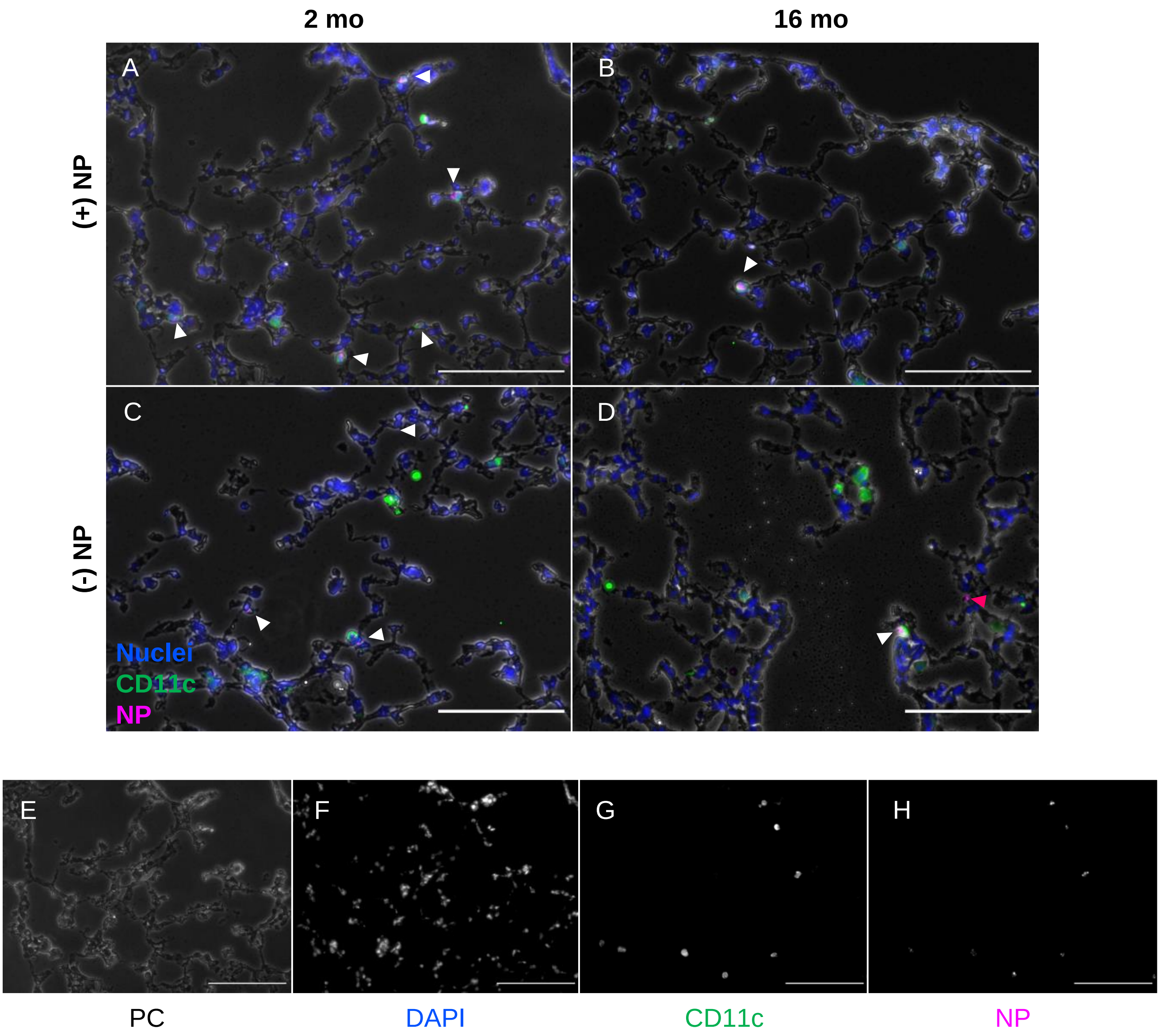


**Supplemental Figure S10: BALF cell replicates and image breakdown.** Nanoparticle-dosed whole lung tissue isolated from 2- and 16-month mice. Additional representative stained images identify nuclei Phase contrast (PC) as background, (DAPI; blue), CD11c (green), and NP uptake (pink). A.) 2-months (+)NP; B.) 2-months (-)NP; C.) 16-months (+)NP; d.) 16-months (-)NP. E-H.) Representative breakdown of composite image including PC (E), Nuclei (F), CD11c (G), and NPs (H). Images were taken using Biotek Cytation 5 Multimode Imager and individually manipulated for viewing of nanoparticles. Scale bar represents 100 µm. White: $NP^{+}$ and $CD11c^{+}$ cell, Pink arrow: free NP

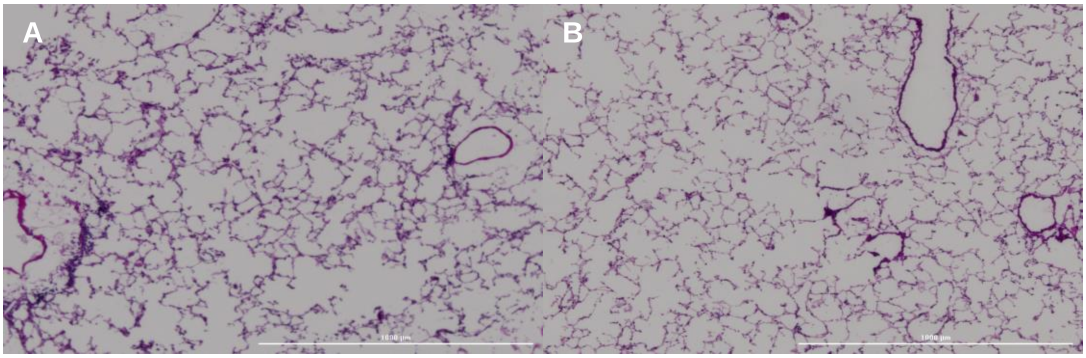


**Supplemental Figure S11: NP uptake inflammation characterization in tissue**. H&E stains were performed on 7 um slices of representative frozen lung tissue from nanoparticle (NP)-dosed mice. Across age and type of NP dosed there were no significant changes in local tissue inflammation. A.) (+) NP B.) (-) NP. Scale bar represents 1000 µm.

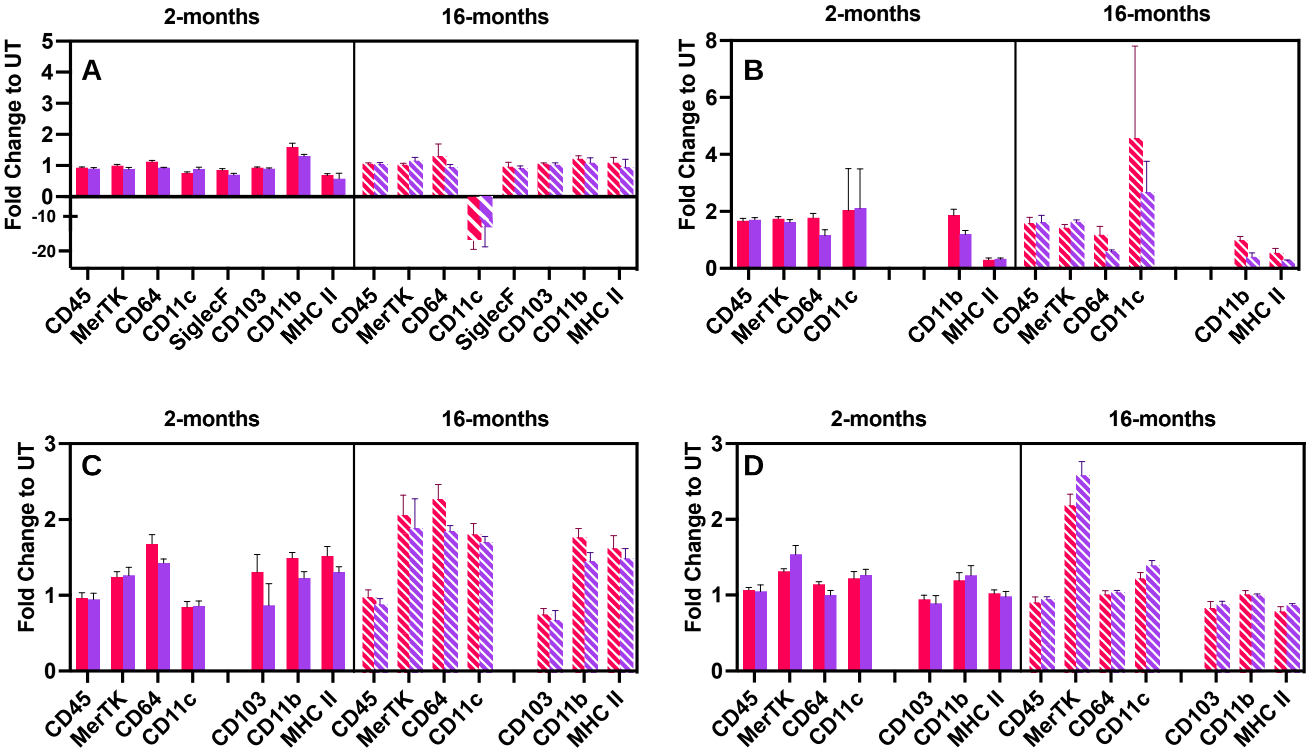


**Supplemental Figure S12: Fold change of markers with NP uptake compared to UT:** Fold change of markers used for flow cytometry of nanoparticle positive cells in comparison to untreated samples from day of measurement. Macrophages (A-B) and dendritic cells (DCs; C-D) were compared across two age groups (2- and 16-months) for 8 markers of interest used to isolate their populations. There were four total groups all together including alveolar macrophages (AMs, A), interstitial macrophages (IMs, B), CD103+ DCs (C), and CD11b+ DCs (D). Values displayed represent mean + standard deviation (n=5) divided by the average value of the untreated specimens based on each age group. Cell types with bar graphs missing represent a marker that was removed due to a lack of expression typically in this cell type.

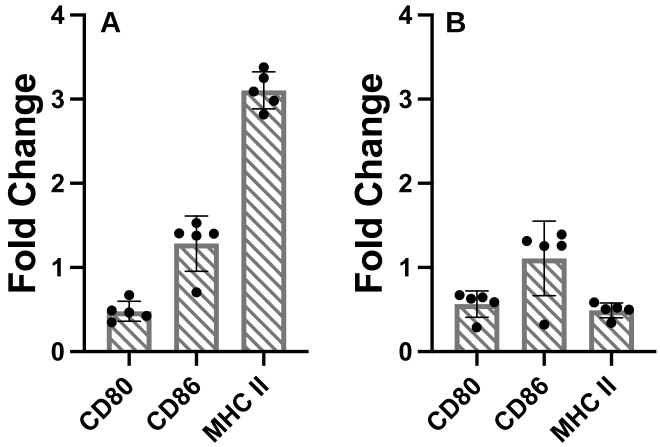


**Supplemental Figure S13: Fold change results for inflammatory profile untreated samples.** MFI (median fluorescence intensity) for isolated alveolar macrophages (A) and dendritic cells (B) was compared for aged (16-month) to young data (2-months) using fold change calculations. Inflammation was characterized via expression of CD80, CD86, and MHC II for these types. Values displayed represent mean + standard deviation (n=5) divided by the average value of the untreated specimens based on each age group. Cell types with bar graphs missing represent a marker that was removed due to a lack of expression typically in this cell type.
